# Single-base resolution methylomes of somatic embryogenesis in *Theobroma cacao* L. reveal epigenome modifications associated with somatic embryo abnormalities

**DOI:** 10.1038/s41598-022-18035-9

**Published:** 2022-09-05

**Authors:** Claudia Garcia, Alex-Alan Furtado de Almeida, Marcio Costa, Dahyana Britto, Fabio Correa, Pedro Mangabeira, Lidiane Silva, Jose Silva, Stefan Royaert, Jean-Philippe Marelli

**Affiliations:** 1 Mars Center for Cocoa Science, Ilhéus, Brazil; 2 Department of Biological Sciences, State University of Santa Cruz, Ilhéus, Brazil; 3 Department of Statistics, Rhodes University, Makhanda, South Africa; 4 Mars Incorporate, Davis, USA

**Keywords:** Biotechnology, Genetics

## Abstract

Propagation by somatic embryogenesis in *Theobroma cacao* has some issues to be solved, as many morphologically abnormal somatic embryos that do not germinate into plants are frequently observed, thus hampering plant production on a commercial scale. For the first time the methylome landscape of *T. cacao* somatic embryogenesis was examined, using whole-genome bisulfite sequencing technique, with the aim to understand the epigenetic basis of somatic embryo abnormalities*.* We identified 873 differentially methylated genes (DMGs) in the CpG context between zygotic embryos, normal and abnormal somatic embryos, with important roles in development, programmed cell death, oxidative stress, and hypoxia induction, which can help to explain the morphological abnormalities of somatic embryos. We also identified the role of ethylene and its precursor 1-aminocyclopropane-1-carboxylate in several biological processes, such as hypoxia induction, cell differentiation and cell polarity, that could be associated to the development of abnormal somatic embryos. The biological processes and the hypothesis of ethylene and its precursor involvement in the somatic embryo abnormalities in cacao are discussed.

## Introduction

*Theobroma cacao* L. (2n = 20) is a preferentially allogamous plant, classified in the Malvaceae family. It has been grown in the lowlands of tropical regions (South and Central America, West Africa and South East Asia) with social and economic importance^1^. Cacao beans are used as raw material to produce cocoa powder, liquor and cocoa butter, which are the main ingredients of chocolate, and to produce additives for pharmaceuticals^2,3^. Despite its importance, cacao propagation has been done by traditional methods with sometimes low efficiencies, such as grafting and rooted cuttings, or by the seedlings generating genetic depression^4^. Other propagation technologies, such as somatic embryogenesis, have been developed with the aim to obtain plants with genetic uniformity^5^. Despite the tremendous efforts to develop a viable somatic embryogenesis protocol, there are still some issues to be solved, including the main one related to the high frequencies of abnormal cacao embryos that cannot be converted into normal plantlets^6^.

Embryogenesis in plants can take place sexually (zygotic embryogenesis) or asexually (apomixis). Zygotic embryogenesis starts with double fertilization of the male and female gametes^7^. Zygotic embryos (ZE) go through different stages of development, i.e., globular, heart, torpedo and cotyledonal in dicotyledonous plants versus, globular, scutellar and coleoptilar stages in monocotyledonous plants; and early and late embryogenesis in gymnosperm plants^8^. Asexual embryogenesis, on the other hand is a complicated process that can be induced naturally (gametophytic or sporophytic) or artificially (somatic embryogenesis), where the stages of development are the same between ZE and somatic embryos (SE)^7^. Although ZE and SE show similarities in the stages of differentiation, their morphologies can be quite different^9^. Besides, ZE are protected by endosperm and SE do not have this structure^8^. Somatic embryos in *T. cacao* have been developed in tissue culture from different explant sources, with the highest success using flower explants (petals and staminodes)^5^. Cacao somatic embryogenesis process is divided in six well defined steps as follows: induction, expression, multiplication, maturation, germination and plant conversion^10^. All these steps are mediated by a complex regulatory network where different genes are regulated at the transcriptional level by both genetic and epigenetic mechanisms^9,11^.

Chromatin structure is affected by epigenetic mechanisms such as histone modifications, small RNAs (sRNAs) and DNA methylation^12^. Chemical modification in the DNA can occur mainly when is acetylated or methylated. Acetylation allows gene transcription, whereas methylation inhibits or enhances it depending where the methyl group is positioned in the gene region^13^. DNA methylation has been widely described as a mechanism implicated in genomic imprinting, repression of gene expression and transposon silencing. DNA methylation in plants occurs when a methyl group is attached to the carbon five of the cytosine. This takes mainly place at cytosines in CpG islands (long repetition of C and G), CHG and CHH (H = A, T or C) sites^14^. DNA methylation profiles may change under variation in environmental conditions that can be stressful for the plants^15^. The position of the methyl group in the gene fulfills important and different functions. If the methyl group is positioned at the regulatory sequences, gene expression is repressed, but methylation also plays an important function when it is placed in the gene body. It has been known that exons are more highly methylated than introns, where methylation in the first exon of the gene blocks gene transcription and methylation in the internal exons does not affect it seriously^16^. On the other hand, methylation in introns is associated with gene transcription enhancement^17^.

DNA methylation plays an important role in early stages of plant development. During embryo growth in *Arabidopsis thaliana,* the methylation profile in CG context is prevalent and remains high constantly during vegetative growth since embryogenesis is initiated, mainly over transposon element (TE) sequences. In contrasts, the methylation in non-CpG contexts is low in TE, especially in the CHH context in post-embryonic development, but during the embryos maturation it gets saturated. The function of DNA methylation in somatic embryogenesis is still no clear but it is related to the morphogenesis activity in of the embryogenic somatic cells, where it acts switching on and off important specific genes in the different stages of embryogenesis, and also in the hypermethylation of TE regions has been associated with the protection of embryogenic cells to the deleterious consequences of TE activity^18,19^.

In somatic embryogenesis, the tissue is subjected to different environmental stimuli, such as plant grow regulators (PGRs), where the explants and the somatic embryos produced in culture undergo stress. A key PGR used in somatic embryogenesis is 2,4-dichlorophenoxyacetic acid (2,4-D), a synthetic auxin that is known as an important cell stressor and has an important role in cell reprogramming through DNA methylation^20^. 2,4-D plays a role in cell polarity and asymmetric cell division that influence changes in the cell morphology, physiology, metabolism and gene expression^21,22^. 2,4-D is also known as a potent inducer of ethylene production in plant tissues^23^. Ethylene is an important phytohormone that works together with jasmonic acid (JA), salicylic acid (SA), abscisic acid (ABA), among others, to regulate several biological processes, including oxidative stress, hypoxia and programmed cell death (PCD)^24^. Furthermore, it is known to act as an important mediator in aerenchyma formation under hypoxic environments^25^.

To understand the epigenetic basis of abnormal somatic embryo production in *T. cacao*, whole-genome bisulfite sequencing (WGBS) was carried out with the aim to identify differentially methylated genes (DMGs), and their functions, among ZE, normal and abnormal SE. In addition, scanning and transmission electron microscopy analyses were carried out to identify morphological and structural differences between ZE and SE. Understanding the main factors that influence the abnormal SE formation could provide guidance towards somatic embryos development processes to improve the quality and efficiency of the plantlets produced. Additionally, our results provide new knowledge in the somatic embryogenesis regulatory network during ZE and SE differentiation in *T. cacao*, providing insights in the basic biology of the somatic and zygotic embryogenesis development.

## Results

### Morphological characterization of somatic embryos

**Figure 1:**
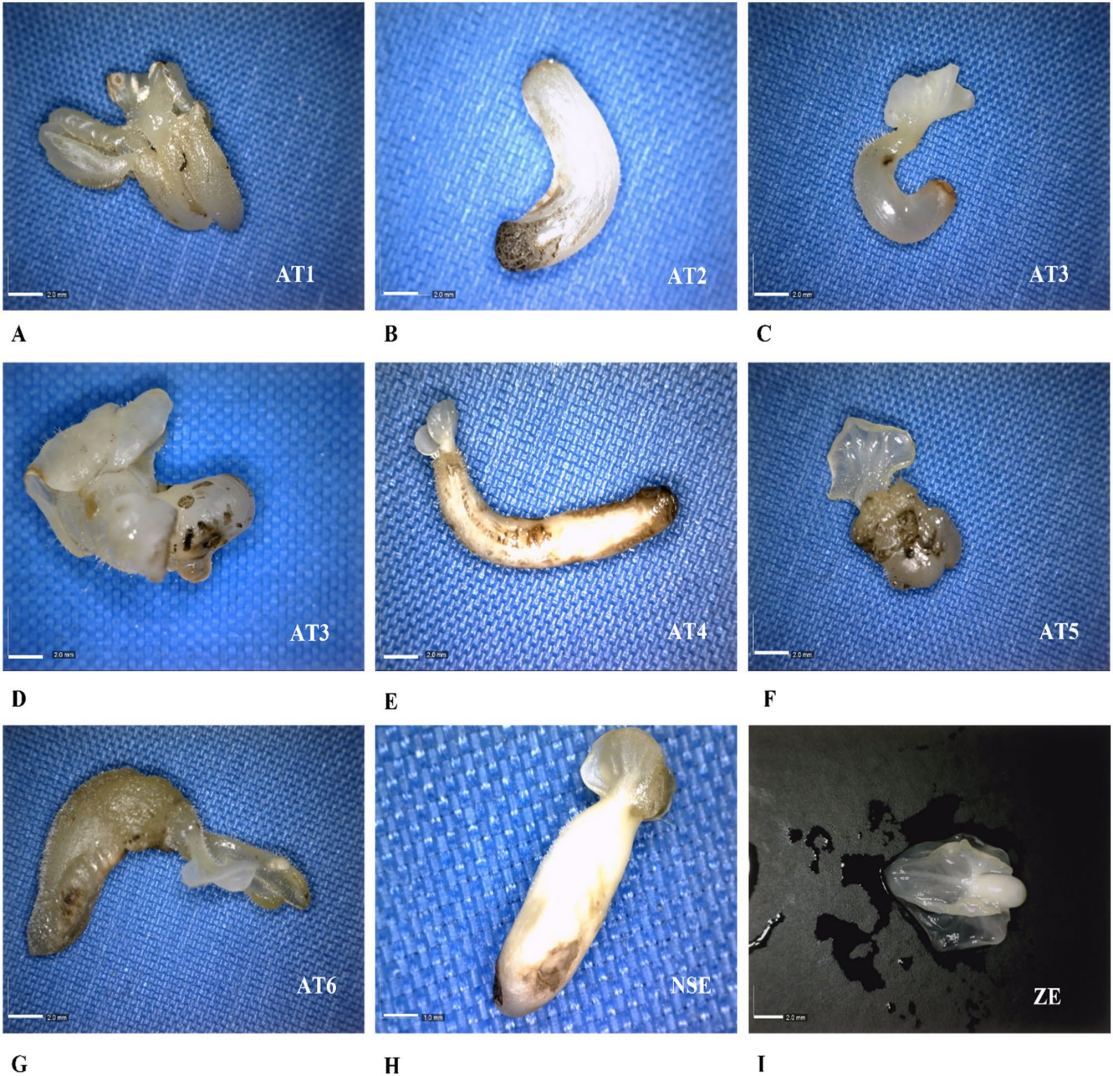
Zygotic and somatic embryos comparation. (**A**) Abnormal type 1 (AT1) has fused more than two embryo axes, (**B**) AT2 are embryos without root and cotyledons formation, (**C**) AT3 are translucent embryos with curved axis, a single cotyledon and without apical shoot formation, (**D**) AT4 are translucent embryos with more than two cotyledons, axis without root and shoot apex formation, (**E**) AT5 are embryos with long and slender axis and poor development of cotyledons without root and shoot formation, (**F**) AT6 are translucent embryos with a single amorphous cotyledons and amorphous axis, (**G**) AT7 are embryos with long and slender cotyledons and curved and amorphous axis, (**H**) NSE normal somatic embryo and (**I**) ZE zygotic embryo in torpedo stage. Scale bar, 2 mm (Zygotic and abnormal somatic embryos); 1 mm (normal somatic embryos).

We first compared the phenotypic characteristics among ZE, normal and abnormal SE at the late torpedo stage to identify the types of abnormalities present in primary SE and secondary SE from CCN 10 (Fig. 1A–I). In a population of 118 primary SE and 530 secondary SE, we were able to identify seven different abnormal types (AT) of embryos. Depending on their morphologies, the AT were classified as AT1–7 (Fig. 1A–G). AT1 has more than two fused embryo axes; AT2 has no root and cotyledon formation; AT3 is translucent with curved axis, single cotyledon and without apical shoot formation; AT4 is translucent with more than two cotyledons, thick axes without root and shoot meristem formation; AT5 contains long and slender axes, poor cotyledon development and without root and shoot meristem formation; AT6 is translucent with an amorphous axes and a single amorphous cotyledons; AT7 is long and slender cotyledons with curved and amorphous axes.

Concerning the primary SE, 18.64% of the embryos were normal type (Fig. 1H) and the other 81.36% were abnormal, where the AT2 was predominant, with frequency of 20.34% from the total population. Of the total secondary SE, on the other hand, only 18.11% were normal and 81.89% were abnormal, with 27.92% of AT2 and 20.75% of AT3 as the main ones (Table 1). The seven anomalous phenotypes of secondary SE were subjected to germination and only 1% of AT2 embryos generated an apical shoot and 4% of AT3 embryos generated axillar shoots and adventitious roots with callus formation in the axis base, while the other SE abnormal types did not germinate (Supplementary Fig. S1A,B).

**Table 1:** Number of normal and abnormal somatic embryos and percentage in primary and secondary somatic embryogenesis.

Type of embryo	Primary somatic embryogenesis		Secondary somatic embryogenesis	
	Number of embryos	Percentage (%)	Number of embryos	Percentage (%)
AT1	24	20.34	49	9.25
AT2	42	35.59	148	27.92
AT3	3	2.54	110	20.75
AT4	12	10.17	22	4.15
AT5	0	0.00	20	3.77
AT6	13	11.02	60	11.32
AT7	2	1.69	25	4.72
Normal embryos	22	18.64	96	18.11
Total	118	100	530	100

### Microscopic analysis

**Figure 2:**
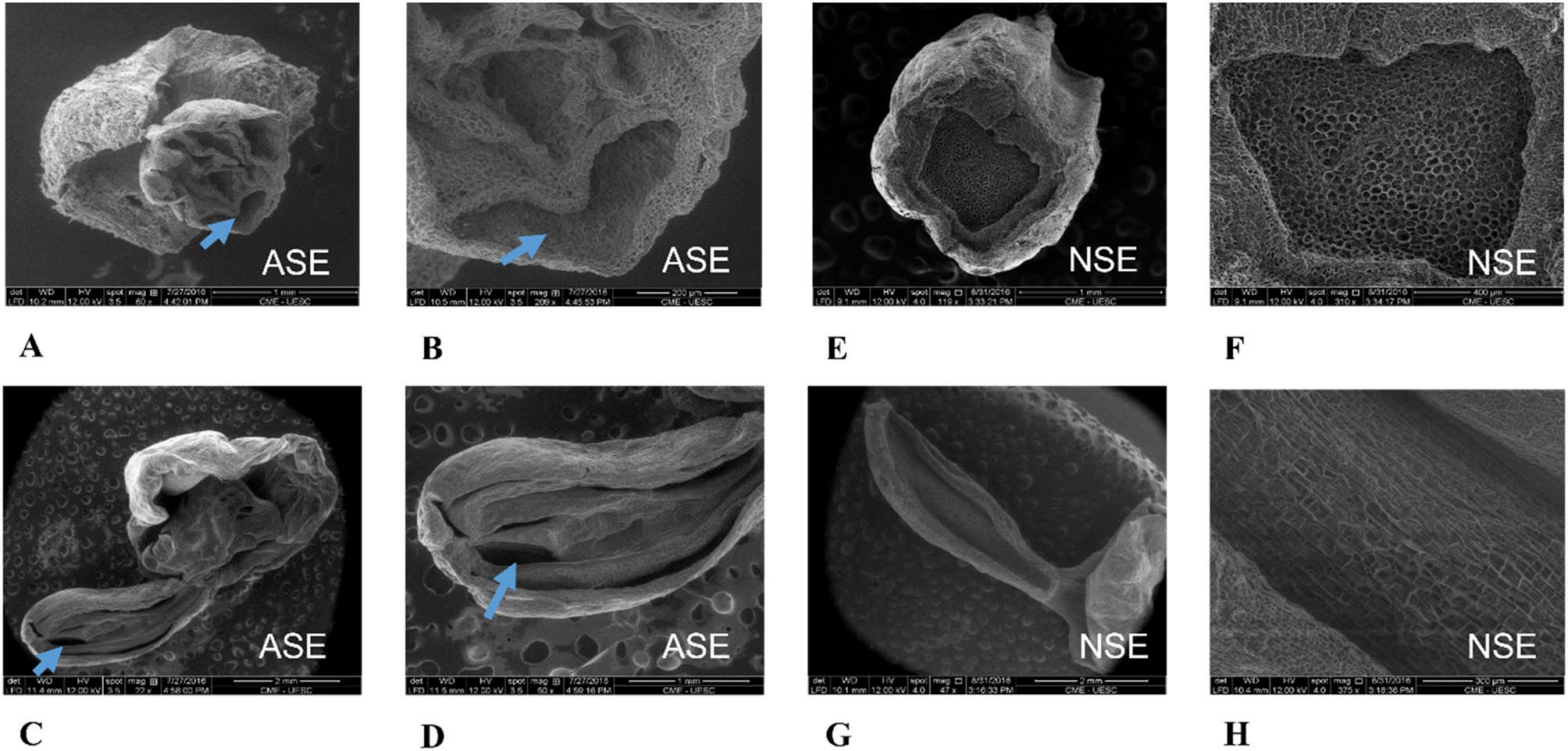
Morphological comparison between normal and abnormal somatic embryos. Scanning electron microscopy of AT4 SE transverse (**A,B**) and longitudinal cuts with detail, where it is observed cavities formation along of their body in these kinds of abnormal SE (**C,D**). Scanning electron microscopy of normal SE transverse (**E,F**) and longitudinal cuts with detail, in difference, normal SE do not have cavities formation in their bodies (**G,H**). Scale bar in the Figures (**A,E,D**) 1 mm; (**C,G**) 2 mm; (**B**) 200 µm; (**F**) 400 µm; (**H**) 300 µm. Blue arrows showing cavities formation in abnormal SE (ASE). *NSE* normal SE.

AT4 embryos were analyzed using scanning electron microscopy (SEM) due to their morphology similarities with normal S. In difference, this type of abnormal SE does not have the capacity to germinate when is compare with other abnormal types like AT2 and AT3 that can germinate producing apical shoots or axillar shoots and adventitious roots respectively (Supplementary Fig. S1) in this way we can compare the different morphological characteristic in the cell layers and internal structures between normal SE and AT4 abnormal SE. Transversal and longitudinal cuts revealed several cavity formations between intercellular spaces along the embryo body (Fig. 2A–D). In contrast, transverse and longitudinal cuts from normal SE showed asymmetric cell divisions and no cavities were formed (Fig. 2E–H). The analysis shows that in an area of 400 mm^2^ there are no cavities in normal SE, but there are on average 3.0 cavities in the same area in AT4 SE, with an average of 217 µm in size.

**Figure 3:**
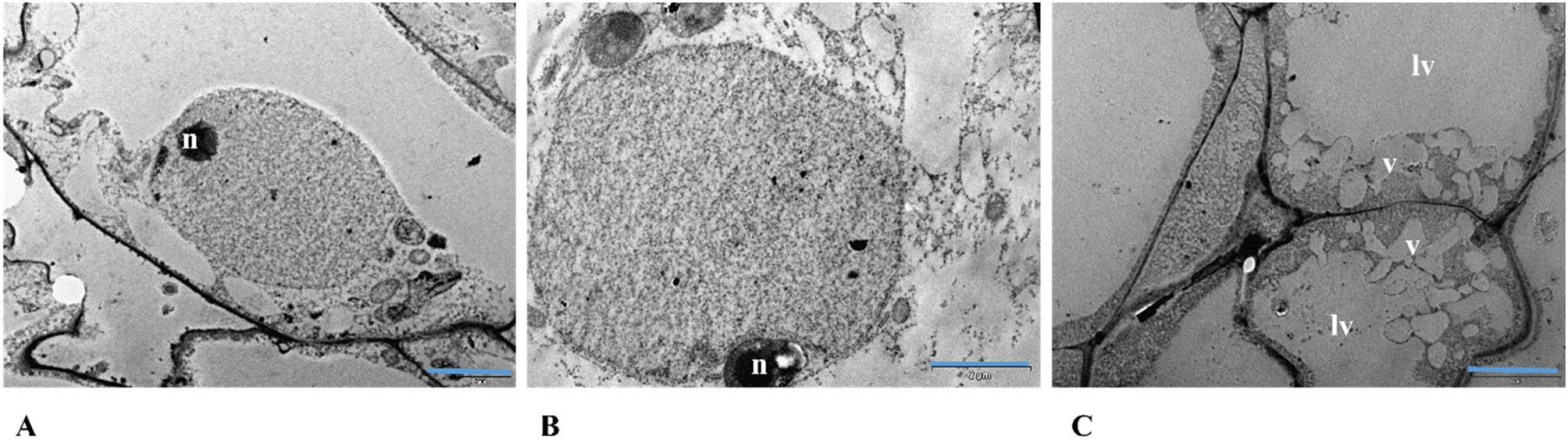
Electron micrographs of zygotic and somatic embryo cells. (**A**) Zygotic, (**B**) normal somatic and (**C**) abnormal somatic cell. Different from zygotic and normal somatic cell, some cells from abnormal SE had a higher number of vesicles, large lytic vacuoles and the nonexsistence of the nucleus. *n* nucleus, *lv* lytic vacuole, *v* vesicles. Scale bar, 2 µm (zygotic and abnormal somatic cells (**A,C**)); 5 µm (normal somatic cell (**B**)).

Transmission electron microscopy (TEM) analysis of ZE and normal SE showed that their cells have nuclei with normal appearances, whereas abnormal SE showed an average of 194 cells without nucleus and lytic vacuoles presence in an area of 42 mm^2^ in its meristematic regions (Fig. 3A–C).

**Figure 4:**
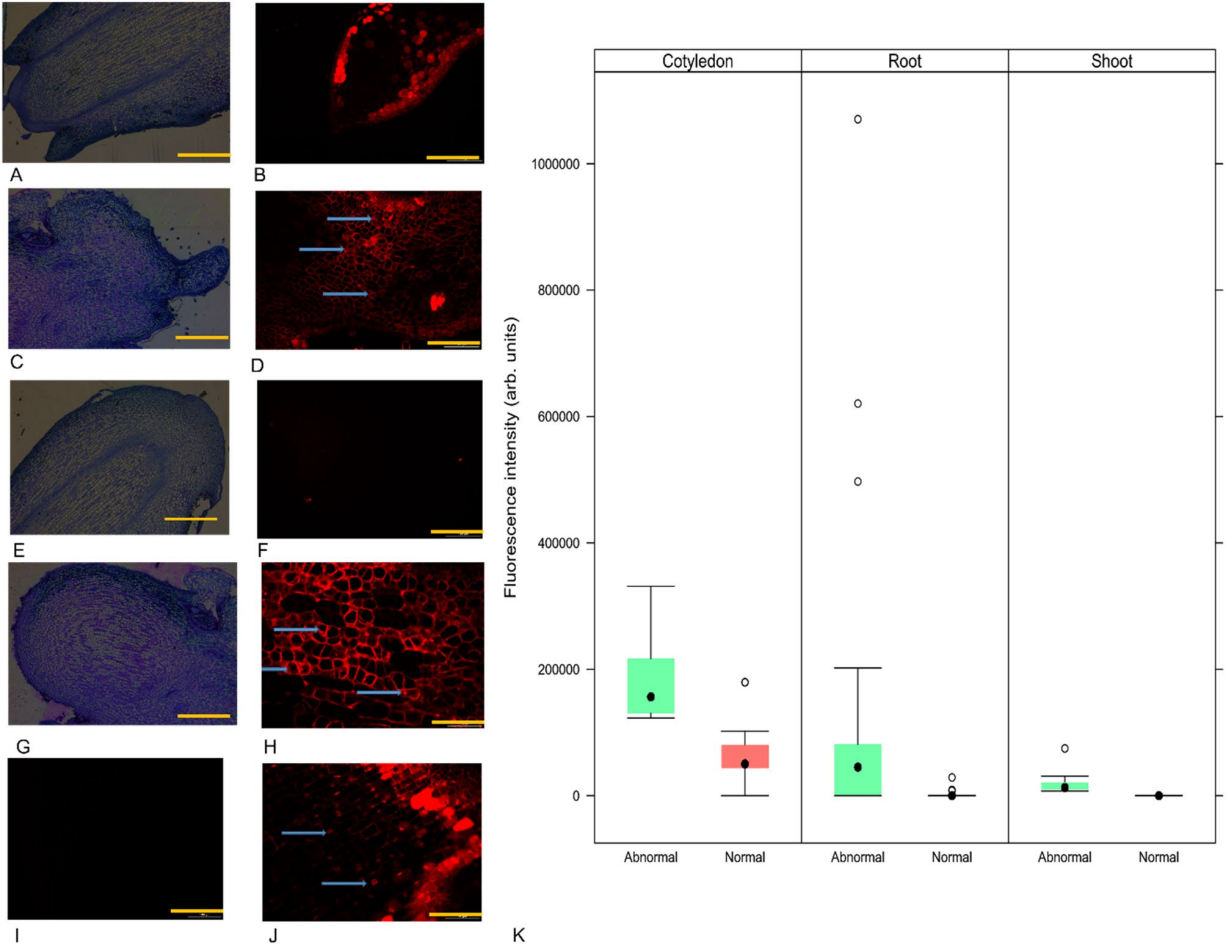
Histological and fluorescence micrographs of normal and abnormal somatic embryos. (**A**) Histological analysis of apical meristem of normal SE. (**B**) Cell death analysis in apical meristem of normal SE, where there is no presence of fluorescence stained in meristematic cells, but superficial cell layers in cotyledons show fluorescence. (**C**) Histological analysis of apical meristem of abnormal SE. (**D**) Cell death analysis in cotyledon and apical meristem of abnormal SE, the nucleus in meristematic and cotyledon’s regions are highly fluorescence stained. (**E**) Histological analysis of root meristem of normal SE. (**F**) Cell death analysis in root meristem of normal SE, where there are no cells-stained fluorescence. (**G**) Histological analysis of root meristem of abnormal SE. (**H**) Cell death analysis in root meristem of abnormal SE shows nucleus-stained red fluorescence. (**I**) Negative control of TUNEL reaction of NSE. (**J**) Positive control of TUNEL reaction of ASE. (**K**) Cell death detection through fluorescence analysis using the TUNEL reaction in normal and abnormal SE. Fluorescence intensity calculated in cells in normal and abnormal SE with cell death, where abnormal SE showed the high values in cotyledons and root meristem cells compared with normal SE. Scale bar, 400 µm (histological micrographs (**A,C,G,E**)); 100 µm (fluorescence micrographs (**B,D,F,H,I**)); 50 µm (Fluorescence micrograph (**J**)). Arrows in blue color are showing apoptotic nuclei inside the cells-stained fluorescence. In figure (**K**) error bars are 95% confidence interval, and outliers are shown as transparent dots.

Normal and abnormal SE exhibited PCD with some differences (Fig. 4A–H). Red fluorescence color in abnormal SE was more intense than in normal SE in the fragmented DNA inside cells (Fig. 4A–H), it is typically characteristic of positive reaction to the kit “In situ” cell death detection fluorescein, product of cell death. While abnormal SE showed cell death in apical and root meristems (Fig. 4C,D,G,H respectively), normal SE showed cell death only in the epidermal layer in cotyledons (Fig. 4A,B) and no cell death in apical and root meristems was detected (Fig. 4E,F).

Normal SE showed an average of 97 cells with PCD in an area of 34.72 mm^2^ in its cotyledons. On the other hand, abnormal SE showed in its cotyledons an average of 195 cells with PCD in the same area. Statistical analysis showed significant differences with p-values < 0.05 in fluorescence intensity in cells of cotyledon, root and shoot regions between normal and abnormal SE. Cotyledons in abnormal SE showed the highest fluorescence intensity, with an average of 2.E+05 arbitrary (arb.) units (Fig. 4I) compared with cells of cotyledons in normal SE, which showed a fluorescence intensity of 6.E+04 arb. units (Fig. 4I). Cells in root meristem region of abnormal SE showed a fluorescence intensity with an average of 1.E+05 arb. units (Fig. 4I), whereas cells in the same region of normal SE showed a fluorescence intensity of 2.E+03 arb. units (Fig. 4I). Cells in the shoot meristem region of normal SE were not stained in red fluorescence, showing no cellular death, but the same region in abnormal SE showed a fluorescence intensity of 2.E + 04 arb. units (Fig. 4I).

### Whole-genome DNA methylation maps of zygotic and somatic embryos

Unbiased DNA methylation maps were generated from a set of mixed embryos samples from ZE, normal and abnormal SE by WGBS. We sequenced a total of 57.2 Gb from ZE, 74.1 Gb from normal SE and 72.5 Gb from abnormal SE. The accession number for the three WGBS is PRJNA857502 in the National Center for Biotechnology Information (NCBI), https://www.ncbi.nlm.nih.gov/sra/PRJNA857502. From the total base pairs, we mapped from each sample 86.14%, 67.08% and 68.30% of sites with methylation in ZE, normal and abnormal SE, respectively, with Phred quality score over 30, where the CpG content in percent (%) is 21.04%, 24.88% and 24.3% in each sample, respectively (Supplementary Table S1). After trimming process to eliminate adapter sequences and bases with low quality from each read, we generated a total of 53.8 Gb (ZE), 57.1 Gb (normal SE) and 54.6 Gb (abnormal SE), which were aligned to the *Cacao_Matina1-6* genome sequence used as reference genome. In the basic statistics of the alignment for ACGT content by chromosome, 86.58–104.03% were covered by at least one unambiguously mapped read with Phred quality score over 30 and standard deviation between 322.33 and 452.18, resulting in an overall average sequencing depth of 30-fold (Supplementary Table S2).

For methylation calling we used a total read of 203,071,419 from ZE, 263,500,094 from normal SE and 245,099,294 from anormal SE (Supplementary Table S3). Methylation calling was different between the contexts. From the total sequencing coverage of CpG, CHG and CHH contexts across all the genome, only 50.14–53.95% (CpG), 28.23–31.58% (CHG) and 5.34–9.91% (CHH) were methylated. Normal SE showed the highest methylation percentages over the three contexts. By contrast, ZE showed the lowest and abnormal SE the intermediate values (Supplementary Table S4).

A total of 7,391,277 CpG, 10,706,841 CHG and 66,034,574 CHH methylation sites were identified in this study. For all the contexts (CpG, CHG, and CHH), the lower sequencing depth cut-off was10. 7,361,253 CpG, 10,679,910 CHG and 65,894,628 CHH methylation sites to be analyzed. In the CpG context we found a binomial distribution in the level of methylation for most of the data (Supplementary Fig. S2). ZE methylation landscape showed that 35.02% of the CpG were with DNA methylation levels between 0.0 and 0.1(low methylation values) and 35.82% were with values between 0.9 and 1.0 (high methylation values). For normal SE, 36.33% of the CpG sites were low methylated and 46.11% were high methylated. Finally, for abnormal SE 35.86% of CpG sites were low methylated and 43.43% were high methylated. These results show that normal and abnormal SE had a higher percentage of high levels of methylated CpG sites in comparison with ZE.

The CHG and CHH contexts were analyzed as bimodal and right-skewed distribution, respectively (Supplementary Figs. S3, S4). The CHG context in ZE, normal and abnormal SE samples showed the maximum data as low methylated, with values between 0.0 and 0.1, and with frequencies of 60.11%, 60.15% and 60.08% respectively. CHH context analysis showed methylation levels between 0.0 and 0.1 in ZE, normal and abnormal SE, with frequencies of 82.42%, 71.85% and 74.65%, respectively. The degree of reproducibility between samples, the normal distribution between samples and the density plot to compare distribution of methylation ratio before and after normalization were analyzed using Pearson’s correlation (Range: − 1 ≤ r ≤ 1), box plot and density plot, respectively (Supplementary Figs. S5A–C, S6A–C, S7A–C).

### DNA methylation dynamics in non-CpG and CpG contexts

**Figure 5:**
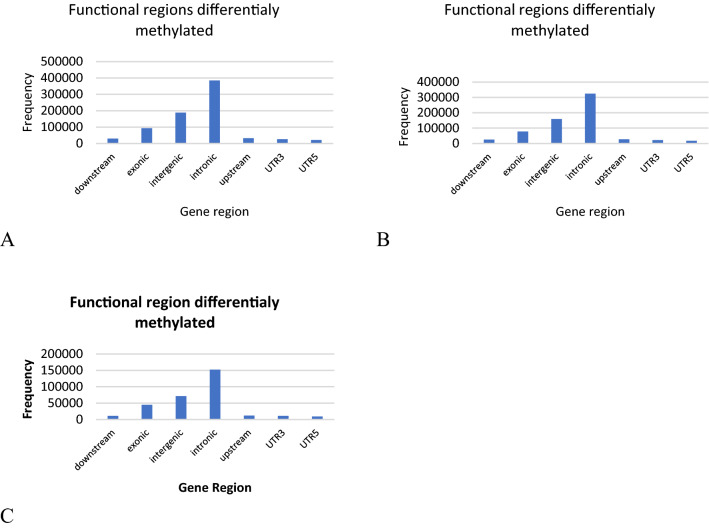
Functional regions differentially methylated analysis in CHG context. (**A**) Frequency histogram representing the functional regions differentially methylated between ZE and normal SE, (**B**) ZE and abnormal SE and (**C**) normal and abnormal SE, where intronic and intergenic regions have the highest frequencies.

Analyzing the CHG context ZE, normal SE and abnormal SE, the hierarchical clustering the Euclidian distances showed that normal and abnormal SE are grouped in the same cluster and ZE are grouped in a different cluster (Supplementary Fig. S8A) indicating that normal and abnormal SE have similar DNA methylation profiles while the DNA methylome of ZE is different. The frequency histograms represent the distribution of differentially methylated regions between samples, where the highest frequencies of the data are grouped to the left showing values with levels of methylation between 0.0 and 0.1 for ZE and normal SE (Supplementary Fig. S8B), ZE and abnormal SE (Supplementary Fig. S8C) and normal and abnormal SE (Supplementary Fig. S8D), indicating that the regions in the CHG context in the DNA methylome are mainly sparsely methylated. The analysis regarding the functional regions differentially methylated in the CHG context showed that introns and intergenic regions are the regions differentially methylated with the highest frequencies when compared to the DNA methylomes between ZE and normal SE (Fig. 5A), between ZE and abnormal SE (Fig. 5B) and between normal and abnormal SE (Fig. 5C). Interestingly, the CHH context has the same profile as the CHG context in the DNA methylome of the ZE, normal and abnormal SE. In the CHH context the samples were grouped in the hierarchical clustering similarly as in the CHG context, normal and abnormal SE are together in the same cluster and ZE is grouped in a different cluster than SE. Differentially methylated regions have the lowest levels of methylation in this context, with values between 0.0 and 0.1, and the high frequencies of functional regions differentially methylated between samples are also the introns and intergenic regions (Supplementary Fig. S9A–G).

**Figure 6:**
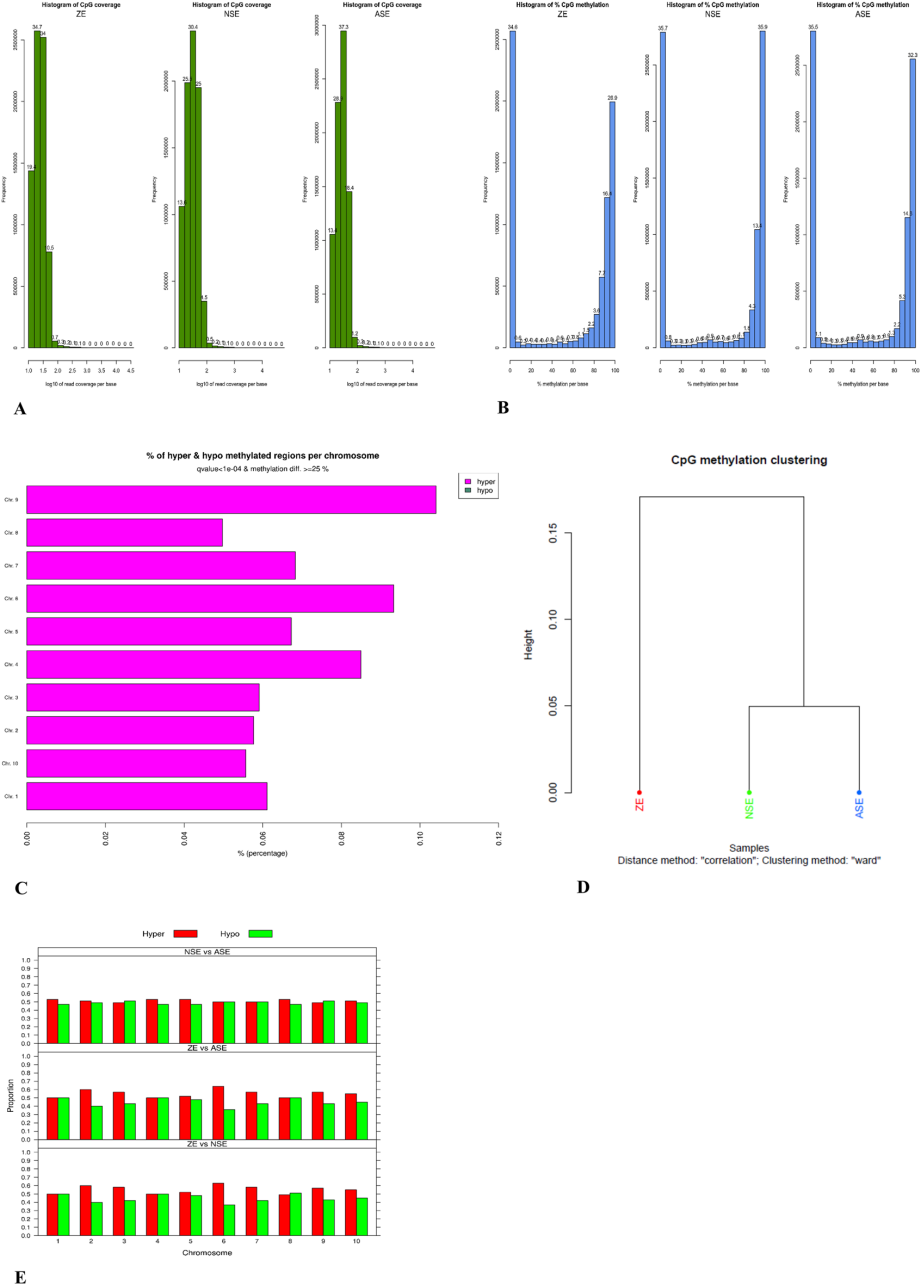
Methylation distribution analysis in the CpG context of the ZE, normal and abnormal SE by WGBS. (**A**) Histogram of CpG coverage for ZE, normal and abnormal SE showing normal distribution for all the samples. (**B**) Histogram of frequencies of CpG percentages in ZE, normal and abnormal SE showing bimodal distribution in all the samples. (**C**) Plot of percentages of significative regions with hypo and hypermethylation in the ten chromosomes comparing the DNA methylomes between ZE, normal and abnormal SE. (**D**) Hierarchical clustering of the samples by ward method, where normal and abnormal SE were grouped in the same cluster while ZE is grouped separately. (**E**) Bar chart of methylation levels proportion between ZE and normal and abnormal SE showing similar proportion for hypomethylation and hypermethylation in whole epigenome. Codes NSE (Normal SE) and ASE (Abnormal SE) in (**E**).

The dynamics in the CpG context are different compared to the CHG and CHH contexts. The methylated CpG region coverage per base followed a normal distribution for ZE, normal and abnormal SE with high frequencies of values between 1 and 2 log 10 of read coverage per base and with percentages between 30.4 and 37.3 of coverage (Fig. 6A). The percentage of methylation in all CpG regions presented a bimodal distribution for ZE, normal and abnormal SE with high frequencies of low methylated regions (0–1%) and highly methylated regions (90–100%) (Fig. 6B). The DNA methylome comparison of ZE, normal and abnormal SE revealed high percentages of CpG regions differentially hypermethylated for all the 10 chromosomes where the chromosomes 4, 6 and 9 showed the highest percentages of regions hypermethylated (Fig. 6C). In the whole CpG context the proportion of regions that are hypo and hyper methylated are quite similar comparing between samples (Fig. 6E). Chromosome 6 shows the highest proportion of hypermethylation comparing ZE with abnormal and normal SE with values superior to 0.5. For the comparison between normal and abnormal SE, chromosomes 4 and 5 presented the highest proportions of hypermethylation, with values superior to 0.6 (Fig. 6E). In the hierarchical clustering CpG methylated regions of normal and abnormal SE are grouped in the same cluster and ZE is grouped in a different cluster (Fig. 6D).

**Figure 7:**
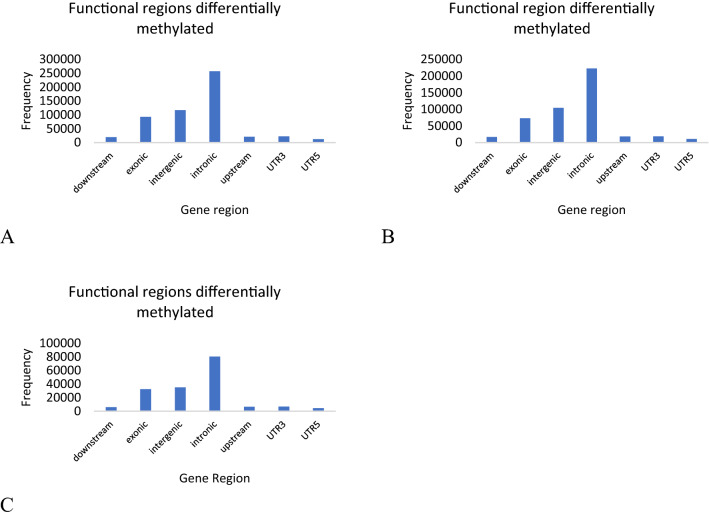
Functional regions differentially methylated analysis in the CpG context. (**A**) Frequency histogram representing the functional regions differentially methylated between ZE and normal SE, (**B**) between ZE and abnormal SE and (**C**) between normal and abnormal SE where intronic, intergenic and exonic regions have the highest frequencies.

From a total of 7,361,253 CpG regions, 545,358 were differentially methylated between normal SE and ZE; 467,448 were differentially methylated between abnormal SE and ZE and 172,607 were differentially methylated between abnormal SE and normal SE. Comparison of the level of methylation between samples was carried out to estimate the level of methylation frequencies. In the histograms (Supplementary Fig. S10A–C) we can observe that there is a bimodal distribution of the levels of methylation for the comparison between ZE and normal SE, between ZE and abnormal SE, and between normal and abnormal SE, respectively. Analyzing the functional regions differentially methylated in the CpG context we found that intronic and intergenic have the highest frequencies as a differentially methylated regions when compared to the DNA methylomes between ZE and normal SE (Fig. 7A) and between ZE and abnormal SE. On the other hand, when compared to the DNA methylomes between normal and abnormal SE (Fig. 7C) intronic regions are also the highest regions hypermethylated, but the intergenic and exonic regions are the second differentially methylated with nearby frequencies. These results show regions differentially methylated in the DNA of ZE, normal and abnormal SE in the CpG contexts, and these regions are strongly hypermethylated, while in the non-CpG context the trend is highest frequencies of hypomethylation in its regions. Secondly, the predominant region with the highest differentially methylated frequencies are the introns in CpG and non-CpG contexts.

### Functional annotation of differentially methylated sequences

Whole genomic sequences from ZE, normal and abnormal SE were analyzed and 873 out of 15,873 (between ZE and normal SE, and between normal and abnormal SE) and 23,957 (between ZE and abnormal SE) were significantly differentially methylated gene regions in CpG context found in this study. These sequences were functionally annotated as retrotransposons, transcriptional factors involved in autophagy, hypoxia cellular response, DNA repair, auxin response, auxin transport, stress response, disease resistance, cell polarity, PCD, cell wall biogenesis, cell transport, cell division, cellular homeostasis, cell signaling, cellular metabolism, and developmental processes such as embryo morphogenesis (Supplementary Tables S5–S8).

**Figure 8:**
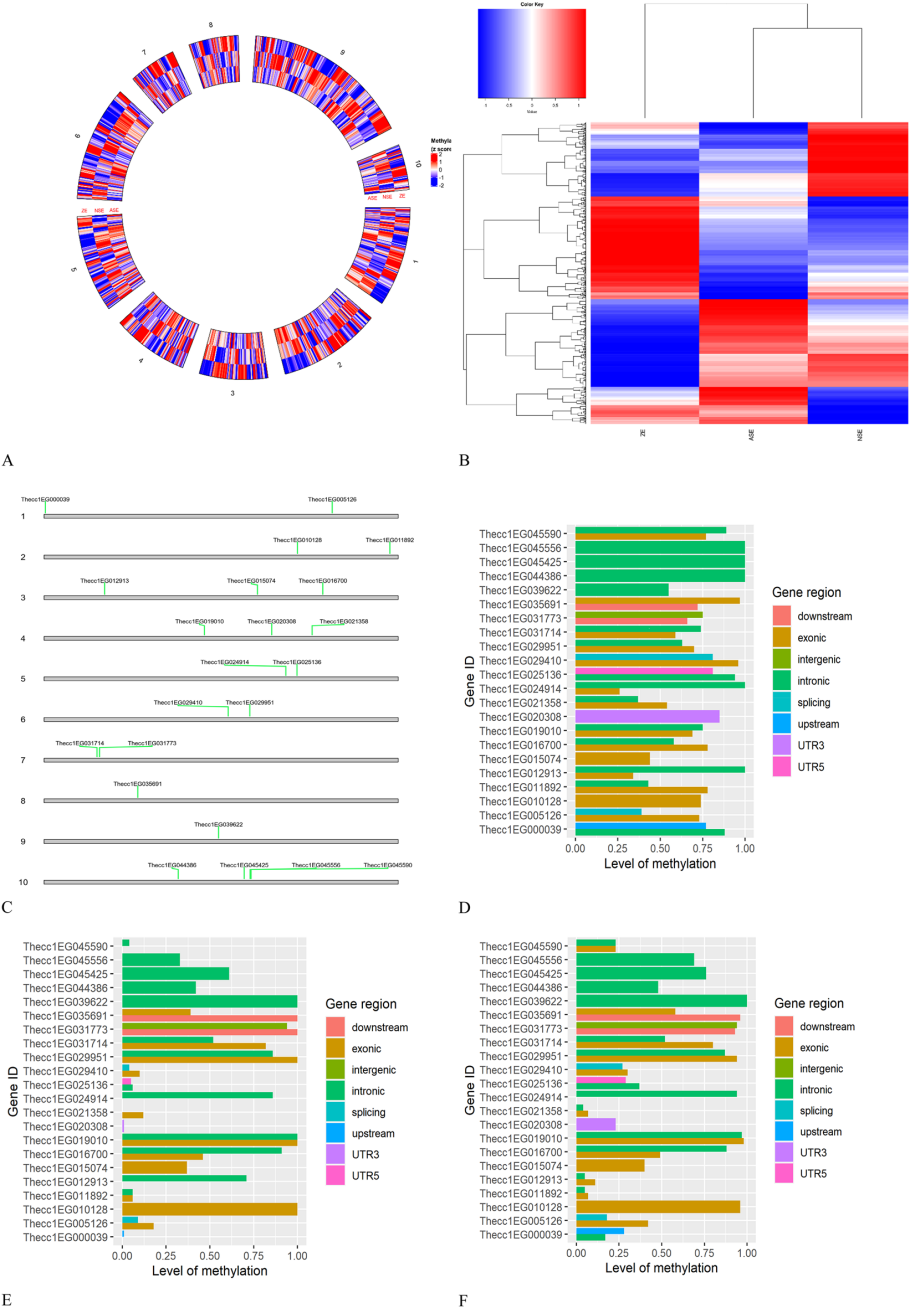
Genes differentially methylated related with SE abnormalities. (**A**) Circular heatmap showing differentially methylated regions in ZE, normal and abnormal SE (*ASE* abnormal SE, *NSE* normal SE) across of ten chromosomes (873 genes). (**B**) Hierarchical heatmap grouping the differentially methylated genes related with similarities between ZE, normal and abnormal SE where 4 groups were formed. For ZE and abnormal SE there were more genes with similar methylation levels, normal SE present high differences when is compare with ZE. (**C**) Gene differentially methylated distribution across of ten chromosomes with important functions in stress response, defense response, auxin transport, cell polarity, PCD, cell wall biogenesis, and embryo shoot and root development. Gene regions differentially methylated between ZE (**D**), normal SE (**E**) and abnormal SE (**F**) with important functions in stress response, defense response, auxin transport, cell polarity, PCD, cell wall biogenesis, and embryo shoot and root development. Color code blue (hypomethylation), white (unmethylated) and red (hypermethylation) in A and B. Protein, chromosome position and function of genes with code (gene ID) are presented in the Table 2. *NSE* normal SE and *ASE* abnormal SE in figures (**A,B**).

Methylation profiles in some regions of these genes were different for ZE and normal and abnormal SE when they are compared across the ten chromosomes (Fig. 8A). Interestingly, ZE and abnormal SE have some regions with similar methylation level, whereas normal SE is generally different from ZE (Fig. 8B). In the heatmap methylated regions are grouped in four hierarchical clusters (Fig. 8B). The first cluster shows genes in ZE and abnormal SE that are hypomethylated, whereas in normal SE these genes are hypermethylated. The second cluster shows genes hypermethylated for ZE and hypomethylated for normal and abnormal SE. The third cluster contains genes in ZE that are hypomethylated and low methylated while in normal SE are hypermethylated and for abnormal SE the same genes are low methylated or not methylated. Finally, in the fourth cluster ZE have genes low and not methylated while normal SE has genes hypomethylated and abnormal SE has genes hypermethylated and low methylated (Fig. 8B). it seems like the gene regulation by the methylation effect is complex and works modulating specific genes in different ways at the same time.

Furthermore, we focused on 22 out of 873 genes that are differentially methylated among ZE, normal and abnormal SE in CpG contexts because they are related to embryos development, PCD, defense and stress response mechanisms probably involved in inducing SE abnormalities as was noted in the microscopy and PCD analysis in this study (Table 2, Fig. 8C). On chromosome 10, the gene Thecc1EG045590 encodes the GroES-like zinc-binding alcohol dehydrogenase family protein with important function in the PCD process. This gene has a high level of methylation in intron and exon regions for ZE (Fig. 8D) but for normal and abnormal SE the methylation level is low (Fig. 8E,F respectively). Those types of embryos were analyzed for PCD, and they presented cells in the meristem zones with DNA damage indicating activity of this gene. Another important gene on the same chromosome is the Thecc1EG044386 that encodes a FASCICLIN-like arabinogalactan protein 16 precursor associated with shoot embryo development. Its intronic region is hypermethylated for ZE (Fig. 8D) but it has low levels of methylation in normal and abnormal SE (Fig. 8E,F respectively). Similarly, Thecc1EG031714 encodes a global transcription factor group E4 on the chromosome 7 involved in embryo root development. ZE has a 0.75 methylation level in its intron and a 0.8 methylation level in his exon, while normal and abnormal SE have a 0.5 methylation level in their intron and 1.0 and 0.9 levels of methylation in their exon, respectively. Methylation in introns is associated with increased gene expression and methylation in exons is associated with gene repression. Intermediated values in the level of methylation of exons in ZE may explain that these embryos are in active development in torpedo stage inside the seed, but high levels of methylation in the same gene region in normal and abnormal SE may associated with development suppression of root meristems where abnormal SE is more repressed than normal SE. This was evaluated in the germination test performed in this work where different types of abnormal SE were submitted to germination and compared with normal SE the abnormal SE does not generate taproot.

**Table 2:** Differentially methylated genes in ZE, normal and abnormal SE with important functions in cellular process implicated in embryos development, stress response, defense response and PCD.

Cacao Gene ID	Chromosome	Position	Context	Protein	Function
Thecc1EG000039	1	168106.0.170565	CG	Auxin efflux carrier family protein	Cellular transport
Thecc1EG005126	1	35923454.0.35927475	CG	Auxin response factor 11	Cellular signaling
Thecc1EG011892	2	42804438.0.42817324	CG	Phosphoenolpyruvate carboxylase 4	Photosynthesis
Thecc1EG010128	2	31421994.0.31432423	CG	WRKY domain class transcription factor	Defense response
Thecc1EG012913	3	7678634.0.7683914	CG	Jasmonate-zim-domain protein 3	Defense response
Thecc1EG016700	3	35110634.0.35118337	CG	Xyloglucanase 113	Cell wall biogenesis
Thecc1EG015074	3	27103765.0.27106730	CG	GroES-like zinc-binding alcohol dehydrogenase family protein	Programmed cell death
Thecc1EG020308	4	28823116.0.28825480	CG	Auxin efflux carrier family protein	Cellular transport
Thecc1EG019010	4	20387381.0.20408383	CG	Auxin transport protein (BIG)	Cellular transport
Thecc1EG021358	4	33815542.0.33819710	CG	Nucleic acid-binding proteins superfamily	Stress response
Thecc1EG024914	5	30,161,616.0.30166159	CG	Nudix hydrolase	Stress response
Thecc1EG025136	5	31557145.0.31566806	CG	Chromatin remodeling factor18	Stress response
Thecc1EG029410	6	22823715.0.22832247	CG	Auxin response factor 6	Cellular signaling
Thecc1EG029951	6	25524446.0.25527828	CG	Bromodomain 4	Stress response
Thecc1EG031773	7	6985775.0.6991143	CG	Phosphoenolpyruvate carboxylase 3	Photosynthesis
Thecc1EG031714	7	6606601.0.6611209	CG	Global transcription factor group E4	Embryo root development
Thecc1EG035691	8	11465349.0.11468083	CG	Peroxidase	Stress response
Thecc1EG039622	9	19300621.0.19303650	CG	Auxin efflux carrier family protein	CELLULAR transport
Thecc1EG044386	10	16464327.0.16469069	CG	FASCICLIN-like arabinogalactan protein 16 precursor	embryo shoot system development
Thecc1EG045425	10	24923124.0.24924516	CG	Peroxidase superfamily protein	Stress response
Thecc1EG045590	10	25845100.0.25847907	CG	GroES-like zinc-binding alcohol dehydrogenase family protein	Programmed cell death
Thecc1EG045556	10	25728048.0.25729425	CG	WRKY DNA-binding protein 70	Defense response

Genes that cod for WRKY domain class transcription factor, jasmonate-zim-domain protein 3 and WRKY DNA-binding protein 70 (Thecc1EG010128, Thecc1EG012913 and Thecc1EG045556 respectively) important in defense response in plants are differentially methylated between the different type of embryos. The Thecc1EG010128 is methylated in the exon for ZE with 0.75 level of methylation, whereas the methylation levels for normal and abnormal SEs are 1.0 and 0.9, respectively. The exon and intron of the gene Thecc1EG012913 are methylated with level of 0.30 and 1.0, respectively for ZE, in normal and abnormal SE the intron is 0.60 and 0.1 respectively, but exon in normal SE is unmethylated, while for abnormal SE exon is 0.1 level of methylation. The intronic region of the gene Thecc1EG045556 is highly methylated for ZE (1.0 level of methylation) and for normal and abnormal SE is 0.30 and 0.70 level of methylation, respectively. Those methylation profiles might be associated with the low stress environment ZE are exposed inside the fruit, in difference normal and abnormal SE are more exposed to inappropriate environment in the in vitro culture conditions.

The different regions of genes that code for the Nucleic acid-binding proteins superfamily (Thecc1EG021358), Nudix hydrolase (Thecc1EG024914), Chromatin remodeling factor18 (Thecc1EG025136) and Peroxidase superfamily protein (Thecc1EG045425) between others are highly methylated for ZE but low methylated for normal and abnormal SE. These genes code for proteins important in stress response. The exonic region of Thecc1EG021358 and Thecc1EG024914 are more methylated in ZE than the same regions in normal and abnormal SE. In addition, the intronic regions of Thecc1EG024914, Thecc1EG025136 and Thecc1EG045425 are more methylated in ZE than the same regions in those genes for normal and abnormal SE. Methylation in exons is associated with induction or repression of gene expression depending on the position of the exon, but methylation in intronic region is associated with gene expression induction. The low level of methylation in the exon region of those genes in normal and abnormal SE might be associated with their high stress response to the in vitro culture conditions.

Thecc1EG039622, Thecc1EG020308 and Thecc1EG000039 are genes located on the chromosomes 1, 4 and 9 respectively, and play a role in the codification of Auxin efflux carrier family protein (*PIN* proteins). Methylation levels in ZE are 0.6 in the intronic region of the gene Thecc1EG039622 and 1.0 level of methylation for both normal and abnormal SE in the same region of the gene. ZE have 0.8 level of methylation in the UTR3 region for Thecc1EG020308 gene and in normal and abnormal SE it reached values of methylation levels 0.0 and 0.25, respectively, in the same regions of the gene. ZE have a methylation level of 0.75 in upstream of the transcriptional start site and 0.8 in the intronic region of the gene Thecc1EG000039. This is quite different from the methylation levels of 0.0 and 0.25 in the upstream region, and the levels of 0.0 and 0.1 in the intronic region in normal and abnormal SE. Auxin efflux carrier proteins fulfill important function in cellular transport where ZE seem to be active differently in this function compared to normal and abnormal SE. Another important gene that codes for an auxin transport protein is Thecc1EG019010 which is located on chromosome 4. In ZE, methylation levels are 0.7 in the exons and 0.75 in introns. Interestingly for normal and abnormal SE both regions have a methylation level of 1.0 which might indicate that probably the auxin trafficking in these cells is blocked (Fig. 8D–F).

Regarding cellular signaling the genes Thecc1EG029410 (Auxin response factor 6) and Thecc1EG005126 (Auxin response factor 11) are differently regulated in ZE, normal and abnormal SE. In ZE the Auxin response factor 6 is methylated in its exonic region and upstream of the transcriptional start site with values of 0.8 and 0.9, respectively. In normal SE, the same regions have methylation levels of 0.1 and 0.2 (upstream and exonic regions). In abnormal SE, the methylation levels are 0.25 for both regions. In the Auxin response factor 11, methylation levels are 0.3 in upstream of the transcriptional start site and 0.7 in exon regions in ZE. In normal SE, methylation levels are less than 0.2 for both of regions. In abnormal SE, the methylation level is 0.2 for upstream transcriptional start site region and 0.4 for the exon region (Fig. 8D–F). The high activity of those auxin response factors in normal and abnormal SE probably can occur because they are exposed to the auxin 2,4-D in the culture conditions.

Non-CpG contexts in the embryo cacao epigenome had 15,942 (between ZE and normal SE and normal and abnormal SE) and 19,783 (between ZE and abnormal SE) differentially methylated gene regions in CHG context. In the CHH context, there were 24,658 (between ZE and normal SE), 23,820 (between ZE and abnormal SE) and 22,893 (between normal and abnormal SE) methylated gene regions. All these differentially methylated gene regions are part of genes that code for proteins with functions in autophagy, hypoxia cellular response, DNA repair, auxin response, auxin transport, stress response, disease resistance, cell polarity, PCD, cell wall biogenesis, cell transport, cell division, cellular homeostasis, cell signaling, cellular metabolism, and developmental processes such as embryo morphogenesis as is the case in the CpG context (Supplementary Tables S9–S14). The more interesting thing is that the non-CpG context were rich in regions of retrotransposon differentially methylated between ZE, normal and abnormal SE with values between 905 and 1002 in the CHG context, where 122 are hypermethylated for ZE and 157 are hypomethylated for normal and abnormal SE. In the CHH context we found between 1062 and 1074 retrotransposons differentially methylated being 38 hypermethylated for ZE and 86 and hypomethylated for normal and abnormal SE. In contrast, the CpG context only had between 707 and 945 differentially methylated retrotransposons being 275 hypermethylated for ZE and 268 hypomethylated for normal and abnormal SE (Supplementary Tables S6–S14) where 14 were significantly differentially methylated when the methylome of ZE, normal and abnormal SE are compared (Supplementary Table S5). Another important result in this work is the high number of methylated regions of unknown genes in the CpG context (between 3933 and 5902), CHG context (between 4422 and 5249) and CHH context (between 5815 and 6137) (Supplementary Tables S6–S14).

## Discussion

*Theobroma cacao* somatic embryos produced in in vitro conditions were classified morphologically in normal and abnormal embryos, with seven different morphologies identified for abnormal SE in our work. Abnormal SE were subjected to germination and only AT2 embryos developed an apical meristem. AT3 embryos were able to develop axillar meristems and adventitious roots, but they were not able to develop into normal plants. Abnormalities in somatic embryos of *T. cacao* are frequently found in other works, where the researchers identified somatic embryos with more than two cotyledons, fasciation and fusion of more than two cotyledons, but the behavior of these abnormal SE in germination is not known^26^. The histological and scanning electron microscopy analysis of abnormal SE revealed abnormal cell division in the provascular tissue originating from the provascular initial cells^27^ and some intercellular cavity formation in the embryo body similar to cavity formation through both schizogenous or lysigenous aerenchyma formation in plants when they are exposed to flooding conditions^28^. Schizogenous aerenchyma is formed by cell division and enlargement of intercellular spaces of cortical cells where there is a separation between the adjacent cell files, whereas lysigenous aerenchyma is formed by the collapse or lysis of the cortical cell files via programmed cell death (PCD)^28^.

PCD in plants is induced by biological processes essential for embryo development, formation and maturation of many tissues and stress response to environmental conditions for adaptation or reaction to biotic and abiotic stimuli^29^. PCD in abnormal SE was notoriously higher than in normal SE in this study. The TUNEL analysis showed that there was DNA fragmentation because the cell nuclei stained fluorescent red in the tissue analyzed, mainly in the embryo body in abnormal SE^30^. The TUNEL analysis is characterized for the label of free 3ʹ OH ends in the fragmented genomic DNA with a fluorescein-dUTP or TMR-dUTP (Tetramethylrhodamine-dUPT) that bright red fluorescent at fluorescence microscope. Another indication of PCD in abnormal SE was the presence of lytic vacuoles in their cells and the absence of a nucleus. These signals are characteristic of vacuolar cell death in plants, which can be stimulated by recycling part of their cells during normal development or stress response^31^. In this study we identified genes differentially methylated in ZE, normal and abnormal SE with important functions in PCD, specifically in cell response to hypoxia, calcium signaling and production of peroxidases which is one of the important reactive oxygen species (ROS) key modulator of plant growth and development, and stress adaptation. Those findings can explain the presence of cavities in the embryo body of abnormal SE, a probably product of lysigeny via PCD for lack of oxygen in the in vitro culture environment, inducing the death of the tissue mediated by ROS^28^.

The methylome landscape of zygotic and normal and abnormal SE from *T. cacao* was developed for first time with the aim to identify gene regions differentially methylated and methylation profiles among them using WGBS. For cacao, we found a global distribution of methylation in the three contexts with some specific characteristics, where non-CpG sequences in ZE, normal and abnormal SE were manly hypomethylated in the CHH context, but in the CHG context there were high frequencies of hypomethylation, but few sequences were also highly methylated. Whereas CpG sequences displayed similar frequencies of hypo and hypermethylation, this result has been consistency with other studies, where levels of DNA methylation are higher in the CpG context than in the CHG and CHH contexts in many plants species^32^.

In several studies was reported that methylation in CpG, CHG and CHH contexts is associated with activation or inactivation of transposon elements (TEs) and repetitive DNA sequences^33^. It has been known that TEs and repetitive DNA sequences are mainly located in intergenic regions of heterochromatin where DNA is highly compacted avoiding the transcription of these regions^34^. Intergenic region of the DNA is heavily associated with closed heterochromatin state, in which its compaction is mainly regulated by the level of methylation in the CpG and CHG contexts, and less in the CHH context^35^. In our study we found that the intergenic region is the second most differentially methylated region between ZE, normal and abnormal SE in the three contexts where were identified retrotransposons significantly differentially methylated spread over the chromosomes 3 to 10 in the CpG context and spread over all 10 chromosomes in the CHG and CHH contexts. Mostly of these retrotransposons were hypermethylated in ZE but hypomethylated in normal and abnormal SE. it was reported in many studies that low levels of methylation in retrotransposons can be associate with their activation^36^, as a result, retrotransposons act as mediator to the stress conditions in the in vitro culture of SE, but retrotransposon activation can also induce abnormal phenotypes SE, as it was identified in other works where the activation of TEs is associated with adaptative response to environmental stress and abnormal phenotypes in plants^37,38^.

Methylation in DNA can inhibit or enhance gene expression depending on where the methyl group in placed. Methyl group can be placed in exon or introns in the gene bodies, or in the transcription start sites, which regulated the expression by enhancing or blocking the gene transcription depending where the methyl group position^33^. Our results demonstrated that introns in ZE, normal and abnormal SE are differentially methylated in high frequency followed by intergenic and exonic regions in all the three contexts. Methylation in exons has been linked to splicing where the mature mRNA is formed by removing introns from the transcribed mRNA precursor^39^. Splicing occurs cotranscriptionally when the transcript is still attached to the DNA and a large fraction of introns are removed from the mRNA precursor by RNA polymerase II^40^.

There is not information about methylation in the different gene regions and their function in normal SE development, only few information in plants development. Therefore, it is important to understand the epigenetic mechanism in other organism as mammalians and unicellular. Regarding to the proportion of methylation between exon and intros, the analysis of DNA methylome in humans showed twice density of methylation in exon when is compare with introns^41^. The high CpG sequences quantity in exons influence in the amount of the total CpG methylation content in the methylome, being exons recognized by the RNA polymerase II and the spliceosome machinery from introns in the splicing^39^. In difference of the studies reported above, in our work we identified high frequencies of differentially methylation regions in introns in all of three contexts (CpG, CHG and CHH) when is compared ZE, normal and abnormal SE methylomes, being exons the third gene site more methylated, important finding because those differences have a strong association with the abnormalities in SE and probably influence the abnormal splicing in the abnormal SE phenotypes.

Considerable increase of gene expression is associated to intronic regions, such as the enhancement of ubiquitin C gene expression in humans^42^ and other genes in yeast^43^ where genes with methylation in the 5ʹ splice sites (5ʹ SSs) of intron regions revealed highest expression compared with genes without methylation in the same regions^44^. In plants, introns play an important role in gene regulation as enhancer element^45^. The mechanism of intron-mediated enhancement occurs when introns are located next to the promotor in the transcribed region, thereby increasing the levels of mature mRNA in the cytosol or when the 5′ SSs (highly methylated) of introns are recognized by U1 Spliceosomal RNA (small nuclear RNA) in the alternative splicing, causing a threefold increase in the transcription products^46^.

In mammals, intronic methylation is an important factor in gene expression regulation of cancer, where hypomethylated motifs in the intron region are associated with upregulation of oncogene expression and hypermethylation is associated with downregulation of tumor suppressive expression^47^. In our work, part of the differential methylation of introns among ZE, normal and abnormal SE was probably due to the different phenotypes presented among them. In addition, differences in the methylation profiles of important genes involved in embryo structure development, stress response to the environmental conditions, and the effects of cellular compounds production can be the consequence of abnormalities in SE. A similar observation was mentioned in a study that was made in non-small cell lung cancer, where methylation profiles were different in two types of cell lung cancer and can be divided in two phenotypically distinct subtypes of tumors using methylation profile as a biomarker platform^48^.

The main differentially methylated genes in this work were those involved in oxidative stress, auxin transport, auxin responsive genes, disease resistance, senescence, hypoxia, and developmental processes. Oxidative stress can start when homeostasis in the cells is disrupted^49^. The principal ROS that are crucial inducer of oxidative stress are mainly singlet oxygen (^1^O_2_*), hydroxyl radical (·OH), hydrogen peroxide (H_2_O_2_), superoxide radical (O_2_^·–^) and nitric oxide (NO)^50^. Hydrogen peroxide is one of the major ROS compounds produced in the cells during abiotic and biotic stress conditions^51^. ROS production can increase and accumulate in the cells, creating an imbalance between ROS production and detoxification, thereby generating abnormal chemical signaling^50^.

Another important function of ROS is the direct effect in the biosynthesis, transport, metabolism and signaling of auxin in plants^52^. Genome regions coding for peroxidase superfamily proteins were identified in our work as differentially methylated, it may be associated with the oxidative stress presented in ZE and normal and abnormal SE in different ways due to abiotic stress induced by the environmental conditions. Hypermethylation in intergenic regions of genes that code for peroxidases in ZE and low levels of methylation in normal and abnormal SE (more in abnormal than normal SE) confirmed that ZE have different environmental conditions than normal and abnormal SEs. In theory, low methylation in intergenic regions is associated with TEs activation when plants are under stress conditions. At the same time, we can infer that abnormal SE presented a particular methylation profile regarding to genes involved in oxidative stress, auxin transport, and developmental processes. Microscopy analysis of the abnormal SE showed abnormal apical-radical axial cell divisions and PCD in the apical shoot and root meristem. Compared to normal SE, this indicates a relationship between ROS and their effect in the wrong auxin polar transport in cells of the abnormal SE.

Oxidative stress induces chemical signals such as abscisic acid, ethylene, brassinosteroids, auxin, gibberellic acid, methyl jasmonate, salicylic acid etc.^50^. The genes that codified the proteins precursor of these compounds were identified in our work as differentially methylated between ZE, normal and abnormal SE. One of the most important is ethylene or its precursor 1-aminocyclopropane-1-carboxylate (ACC) since their production can be associated with the use of 2,4-dichlorophenoxyacetic acid (2,4-D) in the culture medium. Ethylene in high concentration can be accumulated, incrementing respiration rate in the tissue and inducing hypoxia in the culture environment^53^. Ethylene is necessary to stimulate hypoxic induction and it can also regulate aerenchyma formation in root tips of maize plants exposed to hypoxic conditions by schizogeny or lysigeny mediated by PCD and dissolution of cells^54^.

ACC the direct precursor of ethylene has a negative effect in *Arabidopsis thaliana,* in both rosette development and hypocotyl growth, and inhibits primary root elongation independently of ethylene perception when it was applied exogenously^55^. In our research we identified in the methylome of normal and abnormal SE the precursor of ethylene (ACC) differentially methylated downstream of transcription start site of the gene Thecc1EG037684 when is compared with the methylome of ZE. This leads us to speculate that SE are exposed to hypoxic environments in in vitro conditions due to ACC acumulation. Hipoxic environment in the tissue culture can affect some cells in the same tissue generating abnormal development of SE induced by ethylene or ACC.

As hypothesis, the provascular tissue abnormalities in abnormal SE might be associated with and interruption of the auxin transport from the shoot meristem throughout the embryo axis to the root meristem due to the significative differential of methylation profiles between ZE, normal and abnormal SE of genes that encode the *PIN* proteins in the chromosomes 1, 4 and 9. As mentioned above, abnormal SE presented a disorganized growing, without polarity between shoot and root meristems, suggesting problems in auxin efflux transportation^56^. The PIN-formed proteins are transporters acting in the efflux of signal molecules as auxin in plants^57^. *PIN* proteins are asymmetrically localized within cells and their polarity determines the direction of intercellular auxin efflux^57^. *PIN* proteins are located in the plasma membrane and they have influence in plant development by mediating auxin efflux through cells compartments^57^. When the gene that encodes the *PIN7* protein is mutated in the *A. thaliana* genome, induced anomalies in zygotic embryos because the auxin efflux and polarity in early embryogenesis is disrupted^56^. Ethylene is an important regulator in auxin influx and efflux by basipetal auxin transportation. Exogenous application of ethylene inhibits the auxin transportation by proteins as *PIN1*, *PIN2*, *PIN3* and *PIN4* in consequence root formation is disrupted in *A. thaliana.* Ethylene was also reported as important hormone that downregulates the genes that encodes the lateral auxin movement in *Zea mays* and it downregulates the genes that encodes root tips and epicotyls formation in *Pisum sativum* confirming that there is a crosstalk between ethylene and *auxin efflux carrier proteins* in cell polarity^58^.

In conclusion, for the first time the methylomes of torpedo ZE and SE in cacao are reported, revealing a unique profile in abnormal SE accompanying the morphologies presented in those embryos. Although abnormalities in SE prevent embryo germination, we identified seven abnormal phenotypes in a SE production of CCN10 genotype where some of them germinated partially but did not convert in normal plants. The methylome landscape of ZE and normal and abnormal SEs are different showing a specific methylation profile for each one. Different methylation levels were found in the three contexts (CpG, CHG and CHH) in ZE, normal and abnormal SE in cacao, CpG and CHG had a bimodal distribution of hypo and hypermethylated regions. We found similar frequencies of hypo and hypermethylation in the CpG context, and higher frequencies of hypomethylation than hypermethylation in the CHG context. In contrast, the CHH context had the highest frequencies of hypomethylation in a right-squeezed distribution.

The main differentially methylated regions in the three contexts were intronic and intergenic regions. Methylation in intronic regions the highest region methylated in this study is in theory associated with gene expression enhancing, and probably in the wrong identification of introns in splicing process to form the mRNA, important finding in our work that might explain the abnormal phenotypes in SE. Intergenic regions were the second highest differentially methylated regions when ZE are compared with normal and abnormal SE, being less methylated in the abnormal ones. Intergenic regions are heavily associated with heterochromatin remodeling where generally TEs placed. The TEs are implicated in the regulation of stress in the tissue culture environment. Stress environment in the tissue culture was reveled in this study because genes related to oxidative stress were found differentially methylated between ZE, normal and abnormal SE. Microscopy analysis of Abnormal SE revealed cavity formation in their axes. In addition, cotyledons, shoots, and roots in abnormal SE had the highest area of fluorescence values due to PCD. Cavity formation in abnormal SE can be associated with lysigeny via vacuolar cell death probably as a response of the cell to the lack of oxygen in tissue culture environment. Hypoxia induction in abnormal SE can be associated with genes that codes for ethylene and its precursor ACC, which were differentially methylated in this work when compared with ZE. Ethylene production in in vitro environment reduces oxygen concentration and helps stimulating hypoxia induction. Differentiation and cellular auxin transportation of abnormal SE could also be influenced by ethylene in the cell environment due to the in vitro culture condition. Ethylene production in tissue culture could be mediated by 2,4-D (the main synthetic auxin used in the SE culture in this work) that in high concentration is accumulated in somatic tissue affecting embryos development by ethylene effect. An in-depth study of the gene expression of a core selection of the 873 genes found differentially methylated in this work would be required to confirm our results.

## Methods

### Plant material

#### Zygotic embryos

Zygotic embryos at the torpedo stage from cacao pods were obtained by controlled hand pollination of CCN 10 (CCN-*Colección Castro Naranjal)* with CCN 51 as pollen donor in the field at the Mars Center for Cocoa Science (MCCS) germplasm collection. Cacao pods were harvested 13 weeks after pollination (before maturation), which corresponds to the later torpedo stage when embryos maturation has not started yet as previously described^8^. A total of 10 g of ZE were extracted from each pod, frozen in liquid nitrogen and stored at − 80 °C for DNA and RNA extraction.

#### Somatic embryos

Primary SE generated from flowers parts (2000 petals) and secondary SE generated from primary SE cotyledons (60 primary SE) of CCN 10 were obtained using the Li et al. (1998) and Maximova et al. protocols respectively with some modifications previously described in Ref.^6^. The somatic embryos in late torpedo stage as formerly described^9^ were classified in normal and abnormal SE depending on different morphologies presented. Ten grams of secondary normal and 10 g of abnormal SE were frozen in liquid nitrogen and stored at − 80 °C for DNA and RNA extraction.

### Morphological characterization of somatic embryos

Morphological evaluation was done with a representative sample of 118 primary SE (produced from 1000 petals as explants) and 530 secondary SE (produced from cotyledons of 20 primary SE as explants) of CCN 10 in late torpedo stage and using as a control zygotic embryos in the same stage of development and following the references ratings previously described^8,59^, The analysis was performed in a digital microscope (model AM7013MZT, Dino-Lite premier brand). The percentage of normal and abnormal SE was calculated counting the number of embryos (normal and abnormal) into the total of embryos present in each sample and multiplying by hundred. In addition, the percentage of conversion of normal and abnormal secondary SE to plantlets was also calculated to assess the viability of embryos and the capacity to generate normal plantlets. Embryo maturation and conversion were performed using the protocol previously described^6^. Embryos were considered as converted when the shoot apex developed and formed radical extension.

### Microscopic analysis

#### Structure analysis of intercellular spaces in somatic embryos by scanning electron microscope (SEM)

Samples of secondary SE (normal and abnormal) in late torpedo stage (three biological replicates) of CCN 10 were collected and fixed in 2.5% glutaraldehyde, in a sodium cacodylate buffer at 0.1 M, pH 6.8, during 12 h. Subsequently, embryos were washed in the same buffer and were dehydrated in an increasing acetone series (50, 60, 70, 80, 90 and 100% each one for 10 min). Immediately after dehydration, the material was taken to a critical point dryer (model CPD 030, BAL-TEC brand), which allowed the full elimination of water in the plant tissue. Afterward, the samples were placed in a metal stub with double-sided tape on it for subsequent metallization in a sputter COATER, (model SCD 050, BAL-TEC brand), followed by subsequent observation in *SEM* (FEI Company, model Quanta 250, Eindhoven, Netherlands) ^60^. The pictures were captured with CCD and Nav-Cam™ cameras and processed by the Scandium Image software (SIS).

#### Ultrastructural analysis of zygotic and somatic embryos by transmission electron microscope (TEM)

Samples of ZE and secondary SE (normal and abnormal) in late torpedo stage (three biological replicates) of CCN 10 were collected and fixed in 3% glutaraldehyde, in a sodium cacodylate buffer at 0.1 M, pH 6.8, during 12 h. The samples were subjected to a series of washes (3 times for 15 min) in a sodium cacodylate buffer at 0.1 M, pH 7.2, and post-fixed in 1% osmium tetroxide, prepared in the same buffer, during 1 h at 4 °C. Next, the samples were washed 3 times in the same sodium cacodylate buffer for 15 min each one and were then dehydrated in an increasing ethanol series (30, 50, 70, 80, and 90% each one for 15 min), followed by two washes in 100% ethanol (each one for 30 min). Shortly after, the samples were soaked in a mixture of 100% ethanol and LR White resin in the proportions of 3:1 (2 h), 1:1 (2 h), 1:3 (overnight), followed by two changes of pure LR White resin every 4 h, always under slow agitation. Afterwards, the samples were placed in gelatin capsules and covered with pure LR White resin. Resin polymerization was completed after 24 h at 60 °C. The ultrathin section (70 nm) were made with a diamond knife, using a Leica ultramicrotome (model UC6, Nussloch, Germany). The cut sections were deposited on copper grids (300 mesh), contrasted with uranyl acetate in aqueous solution for 25 min, and then with lead citrate for 30 min. Subsequently, they were observed in *TEM* MORGAGNI and wide angle Olympus MegaView III camera and the iTEM software (FEI Company, model 268 D, Eindhoven, Netherlands) ^61^.

#### Cell death detection and histological analysis

Cell death was detected using the “In situ” cell death detection fluorescein kit provided by ROCHE. Samples of normal SE and abnormal SE in late torpedo stage of CCN 10 were collected and fixed in FAA solution (Combine 10 parts 37–40% formaldehyde, 70 parts 95% ethanol, 15 parts dH_2_O, and 5 parts acetic acid) during 12 h. The samples were submerged in paraffin and sectioned in slices of 5 µm using Leica ultramicrotome (model UC6, Nussloch, Germany). The tissue sections were pretreated with proteinase K diluted in Tris–HCl 10 mM (20 mg/mL) pH 7.4 during 20 min. After that we followed the manufacturer recommendations. Tissue sections were analyzed in LEICA fluorescence microscope model DM2500 identifying red fluorescence with the Leica Application Suit (LAS X) software (Cells-stained fluorescent red are positive for cell death). The negative control was prepared using normal SE tissue incubate sections with label solution only, instead of TUNEL reaction mixture. The positive control was prepared using the abnormal SE tissue. Sections of abnormal SE were incubated with DNase I (3000 U/mL in 50 mM Tris–HCl, pH 7.5, 1 mg/mL Bovine Serum Albumin (BSA)) for 10 min at 15–25 °C to induce DNA strand breaks, prior to labeling procedure.

Pictures were captured with the QImaging Retiga 4000R monochrome camera, using the Texas Red fluorescent filter (Leica # TX2)**.** Same tissue sections were treated with 1% toluidine in a solution of 0.1 M sodium phosphate and pH 6.8 for histological analysis. Area and intensity of fluorescence were calculated using the ImageJ program (https://imagej.nih.gov/ij/download.html), and the statistical analysis was performed with the software R 4.0.2.

### DNA extraction

Genomic DNA from a population of 200 normal SE, 200 abnormal SE and 200 ZE at the late torpedo stage was extracted using the DNeasy extraction kit Plant Mini Kit (250) from QIAGEN for future methylation analysis. A tissue sample of 100 mg was disrupted in liquid N_2_ to a fine powder using a mortar and pestle, transferred to a 2 µL Eppendorf microtube and DNA immediately extracted, following the kit protocol (DNeasy^®^ Plant Handbook, QIAGEN). The extraction procedure was repeated two times to ensure the DNA quality and quantity. After the second extraction, DNA was precipitated with 1:1 volume of isopropanol and 2 µL of glycogen from a stock of 20 mg/mL for 20 min at − 20 °C. The samples were centrifuged in a bench-top centrifuge (Mikro 220R-V1.20, Hettich Zentrifugen, Germany) at 8000×*g* for 30 min at 20 °C. The supernatant was discarded, the resulting pellet was left to dry for 30 min in environmental conditions. Afterward, the pellet was re-suspended in 50 µL of 100% ethanol. The DNA quality was checked on a 1% agarose gel in 1× TBE (2.5 g/L boric acid, 10.8 g/L Tris base, 4 mL of 0.5 M EDTA, pH 8.0) using a molecular marker (Hi DNA mass Ladder, Invitrogen brand) as reference. DNA quantification was performed using a spectrophotometer Multiskan GO, Thermo Scientific brand by absorbance at a wavelength of 260/230 and 260/280 nm.

### Global DNA methylation by whole-genome bisulfite sequencing

The Whole-Genome Bisulfite Sequencing was performed on the total DNA extracted from two replicates of a set of mixed embryos from each of the three representative samples (normal SE, abnormal SE and ZE), using the protocol to prepare samples for Illumina sequencing platform (WBS for methylation analysis preparing samples protocol (KK8201 & 59104)). For a few minutes, genomic DNA (5 μg) was normalized with unmethylated lambda (λ) DNA 5% (5 ng λ DNA per microgram of genomic DNA, Promega). DNA was fragmented by sonication using a Covaris S220/E220 sonicator, and fragment selection (150–300 bp) was performed with AMPure XP beads (Agencourt Bioscience). Genomic DNA libraries were constructed using the Illumina TruSeq Sample Preparation kit, considering the Illumina’s standard protocol. Repair was performed at the ends of the DNA; at the 3ʹ end of each fragment an adenine was added, and the Illumina TruSeq adapters were also ligated to both ends. After ligation of the adapters, DNA was treated with sodium bisulfite using the EpiTect Bisulfite kit (QIAGEN). The bisulfite conversion was performed two times to ensure a conversion rate over 99%. Seven PCR cycles were carried out to ensure the enrichment of the adaptor-ligated DNA using PfuTurboCx Hot-Start DNA polymerase (Stratagene). Library quality and concentration were checked using the Agilent 2100 Bioanalyzer. Paired-end DNA sequencing (2 × 100 bp) with 30× coverage was then performed using the Illumina HiSeq 2000 platform.

#### Read mapping and estimation of cytosine methylation levels in CpG, CHH and CHG contexts

Before mapping and estimation of cytosine methylation levels, the raw sequence reads were filtered to make the quality check using the FASTQC bioinformatics tool (https://sourceforge.net/projects/fastqc.mirror/) with Phred quality score over 20 at each cycle. Also the adapter sequences were trimmed off the raw sequence reads using Trimmomatic program (http://www.usadellab.org/cms/index.php?page=trimmomatic)^62^. Read mapping was carried out using the BSMAP (http://code.google.com/p/bsmap/) which is based on the SOAP (Short Oligo Alignment Program) against to the Cacao_Matina1-6 (Tcacao_genome_v1.1) genome as a reference (https://www.cacaogenomedb.org/Tcacao_genome_v1/). The only uniquely mapped reads were selected to sort; index and PCR duplicates were removed with SAMBAMBA (v0.5.9). The evaluation of the quality of the alignment data (a BAM file) and the basic statistics of the alignment (ACGT Content, mean and standard coverage by chromosome, insert distribution etc.) was performed with Qualimap 2.2. The methylation ratio of every single cytosine location was called from the mapping results using ‘methylatio.py’ script in BSMAP. The results of the coverage profiles were calculated as the number # of C/effective CT counts for each cytosine in CpG, CHH and CHG contexts^63^.

#### Differential of methylation in CpG, CHH and CHG contexts

DNA methylation ratio values of single cytosine obtained from BSMAP program were used as the original raw data from the three samples (normal SE, abnormal SE and ZE). The methylation ratio values of all samples were normalized using median scaling normalization to compare the methylation level between samples. During data preprocessing, low coverage and high coverage bases were filtered. Lower read cut-off of 10 means that bases with coverage below 10× were discarded because a high enough read coverage will increase the power of the statistical tests. Higher read cut-off of 500 means that bases with more than 500× in each sample were also discarded.

Statistical analysis was performed using the difference of two groups (delta_mean) per comparison pair. The significant results were selected on conditions of |delta_mean|≥ 0.2 with p-value of 0.05. Level of methylation was displayed as hypermethylation when the delta mean ≥ 0.2; and hypomethylation when the delta mean ≤  − 0.2. For significant CpG list, hierarchical clustering analysis was performed to group the similar samples grouped in CpGs, CHGs and CHHs. A cluster analysis was performed considering the correlations between the samples and grouping with Ward’s method^64^. Differential methylation was obtained from the regions significantly different using Logistic regression (p < 0.001). The analyzes were performed with the aid of packages methylKit, complexHeatmap, circlize and annotate using software R 4.0.2.

#### Genomic and functional annotations of CpG, CHH and CHG sites

Each cytosine locus in CpG, CHH and CHG contexts was annotated using the Table Browser tool of the University of California Santa Cruz (UCSC) Genome Browser. Annotation included functional location of each gene (promoter regions which are defined as − 2 kb upstream of the transcription start site, exons and introns, intergenic, and UTRs regions), transcripts identification (ID), gene ID, strand, and sequence context^65^. The gene function was identified by making the alignments with expectation values (*E*-value) < 0.001 with the aim to infer gene homology comparing with others genomes such as *A. thaliana* using the cacao genome database in the Phytozome V13 (https://phytozome-next.jgi.doe.gov/info/Tcacao_v2_1) and the translated-DNA: protein alignments by BLASTX similarity search tool in the National Center for Biotechnology Information (NCBI) databases (https://blast.ncbi.nlm.nih.gov/Blast.cgi?PROGRAM=blastx&PAGE_TYPE=BlastSearch&LINK_LOC=blasthome). The protein metabolic function was identified using the collection databases from Kyoto Encyclopedia of Genes and Genomes (KEGG) (https://www.genome.jp/kegg/).

This research was carried out in Brazil and the plant material used in it was donated from the germplasm collection of the Mars Center for Cacao Science (MCCS), which complies with the relevant national guidelines and the legislation of the Conselho de Gestão do Patrimônio Genético—CGEN permit for access to genetic resources number 02000.000070/2015-05.

## Supplementary Information

Supplementary Information.

Supplementary Table S5.

Supplementary Table S6.

Supplementary Table S7.

Supplementary Table S8.

Supplementary Table S9.

Supplementary Table S10.

Supplementary Table S11.

Supplementary Table S12.

Supplementary Table S13.

Supplementary Table S14.

## Data Availability

The datasets generated and/or analyzed during the current study are available in the [National Center for Biotechnology Information] repository, [https://www.ncbi.nlm.nih.gov/sra/PRJNA857502].
