# Supplementary material for: Single-base resolution methylomes of somatic embryogenesis in Theobroma cacao L. reveal epigenome modifications associated with somatic embryo abnormalities
Source: Sci Rep. 2022 Sep 5;12:15097. doi: 10.1038/s41598-022-18035-9 (PMC9445004; doi:10.1038/s41598-022-18035-9)
Supplement: Supplementary file 1 — Supplementary Information. [file 41598_2022_18035_MOESM1_ESM.docx]

**Supplementary Figures and tables**

Single-Base Resolution Methylomes of Somatic Embryogenesis in *Theobroma Cacao* Reveal Epigenome Modifications Associated with Somatic Embryo Abnormalities


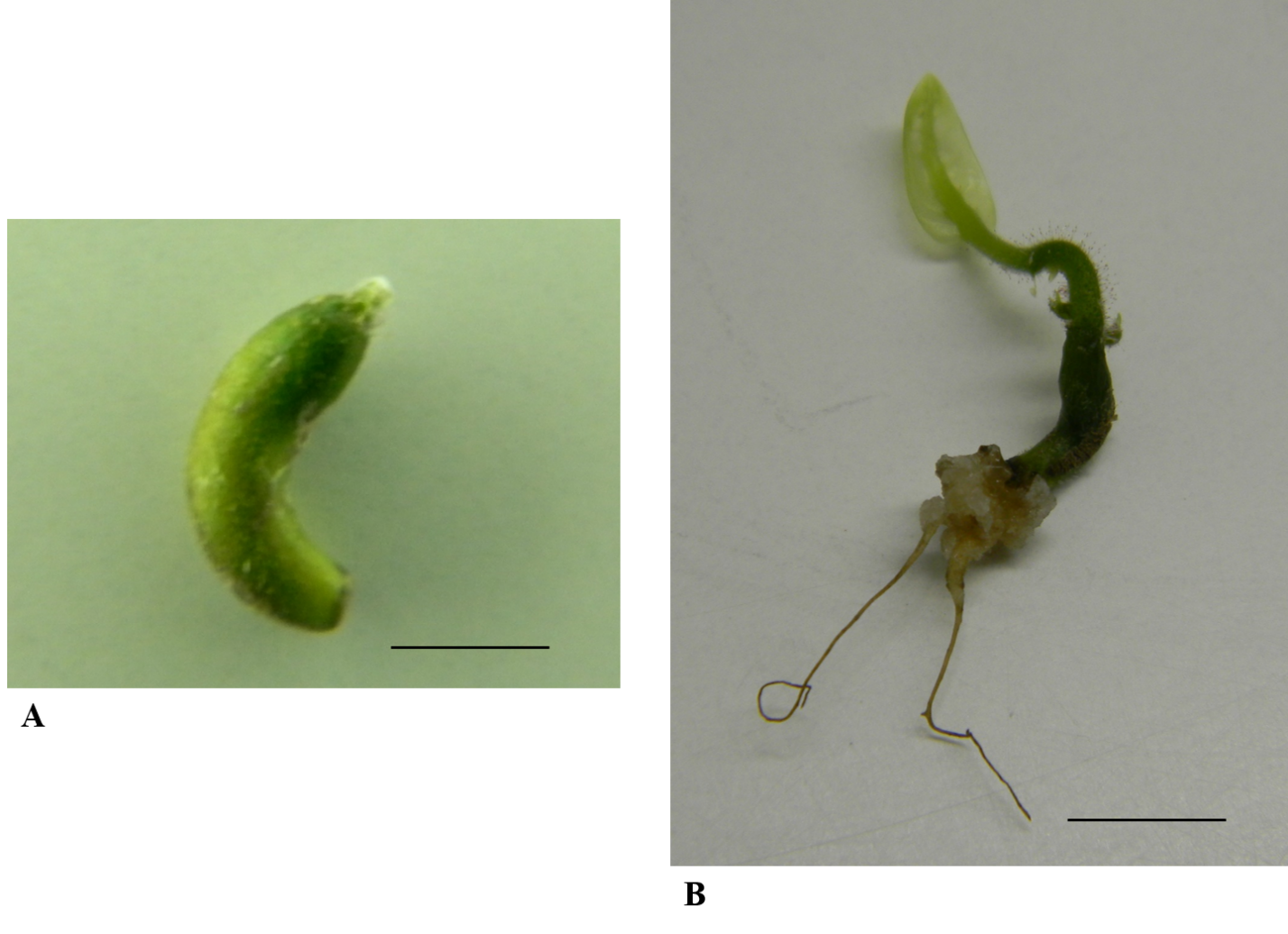


**Supplementary Figure S1.** Abnormal embryos germinated. Abnormal embryo (AT2) with apical shoot (A), AT3 with axillar shoots and adventitious roots with callus formation in the axis base (B). Scale bar 1cm in figures A and B.


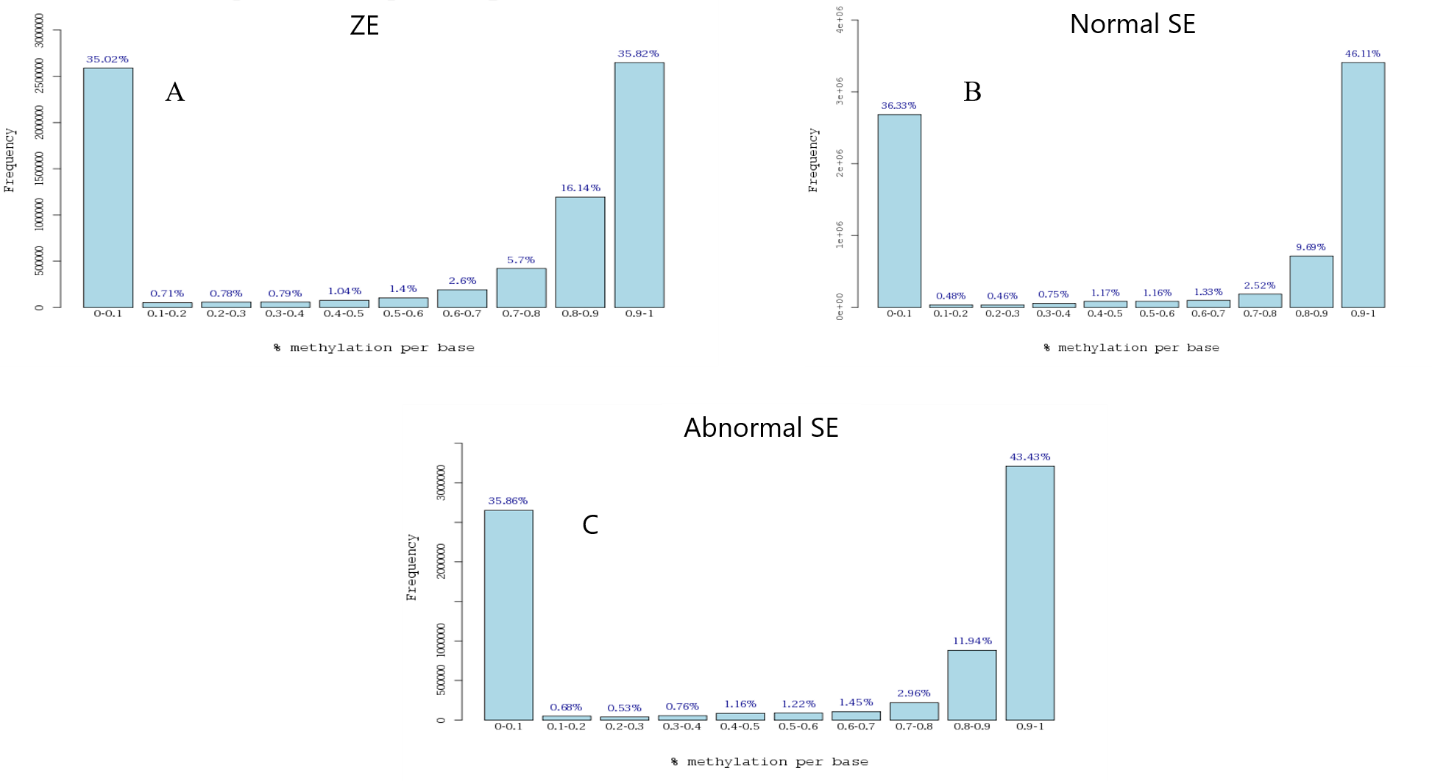


**Supplementary Figure S2**. Histogram of percent methylation distribution and CpG coverage per sample. ZE (A), Normal SE (B) and Abnormal SE (C).


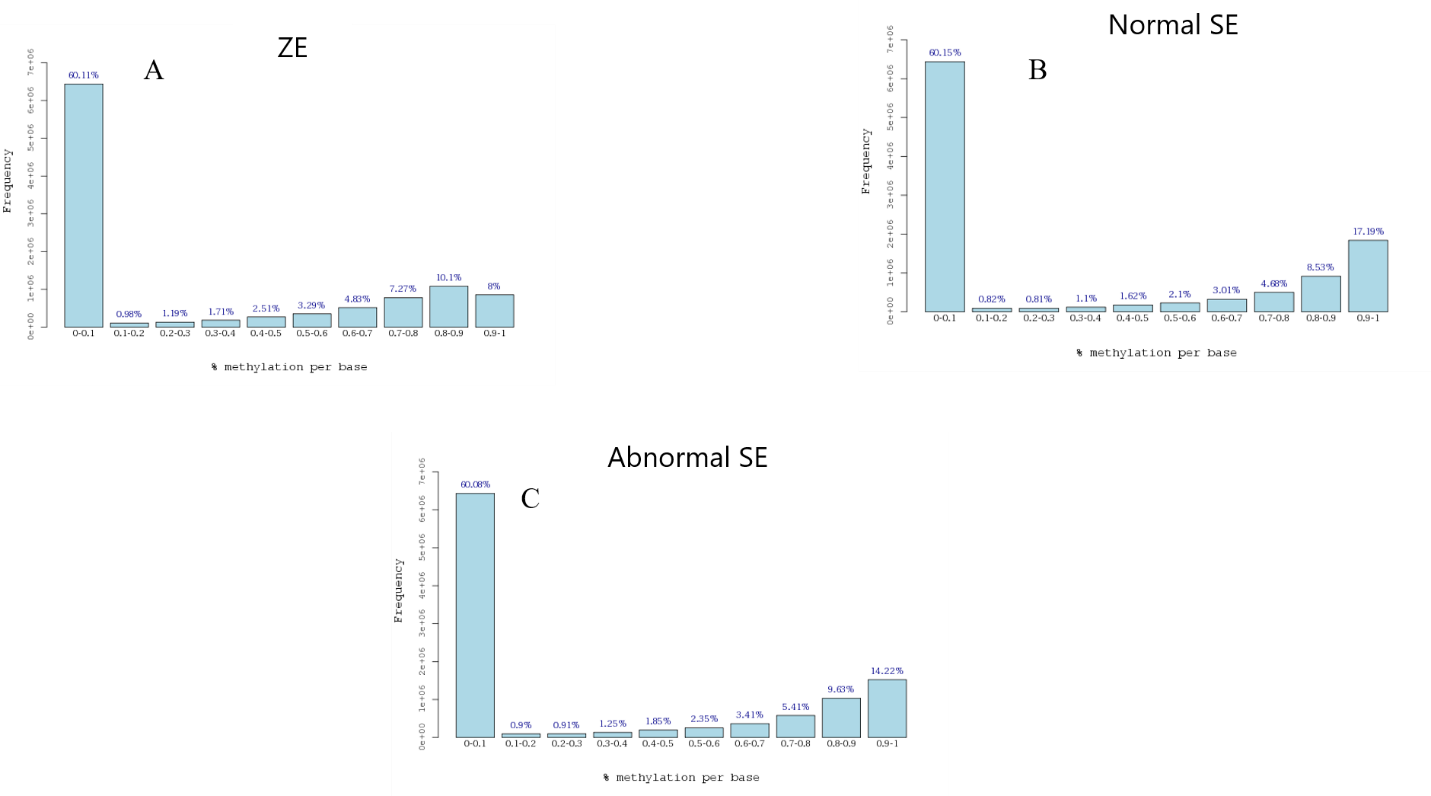


**Supplementary Figure S3**. Histogram of percent methylation distribution and CHG coverage per sample. ZE (A), Normal SE (B) and Abnormal SE (C).


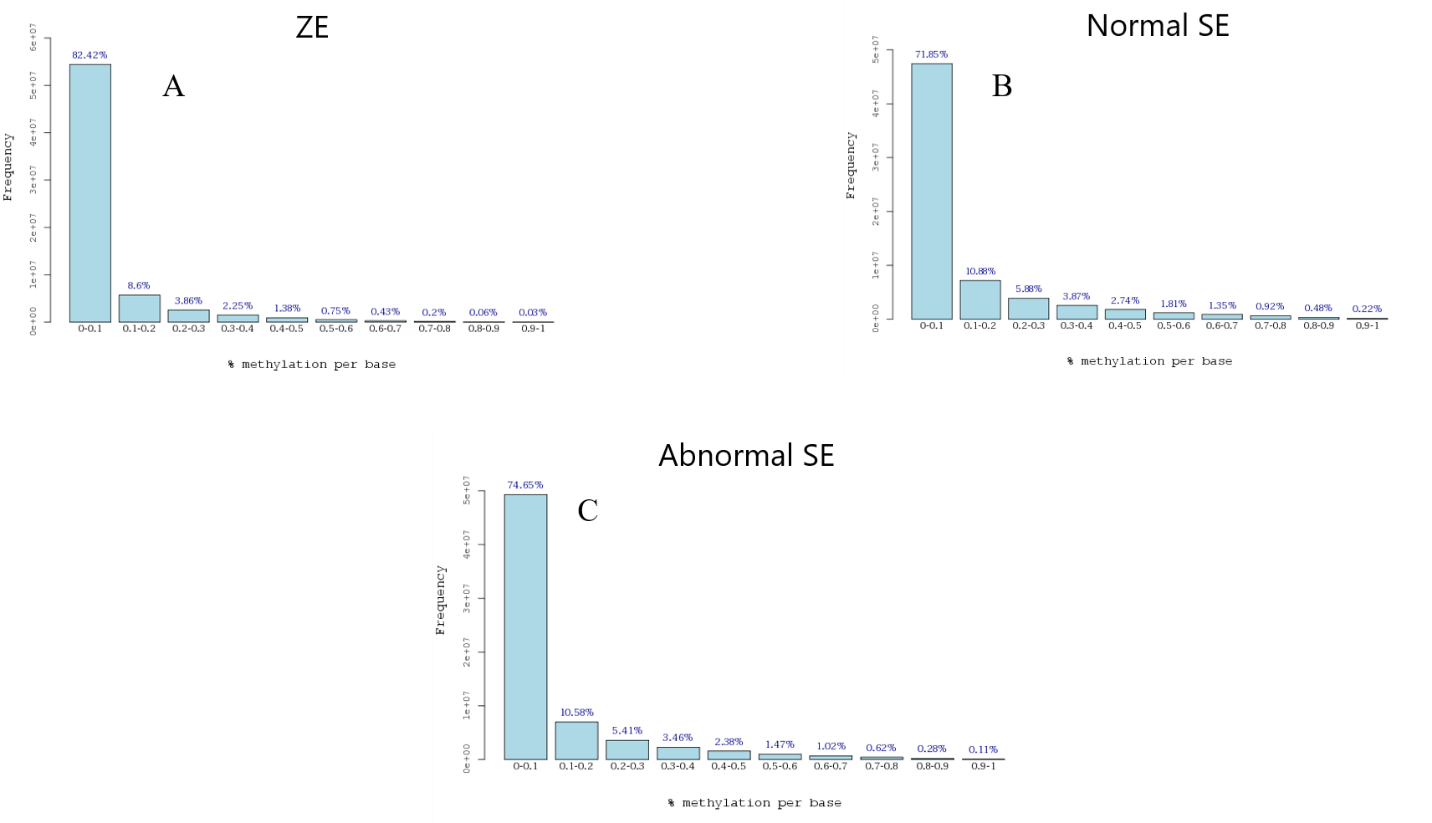


**Supplementary Figure S4**. Histogram of percent methylation distribution and CHH coverage per sample. ZE (A), Normal SE (B) and Abnormal SE (C).


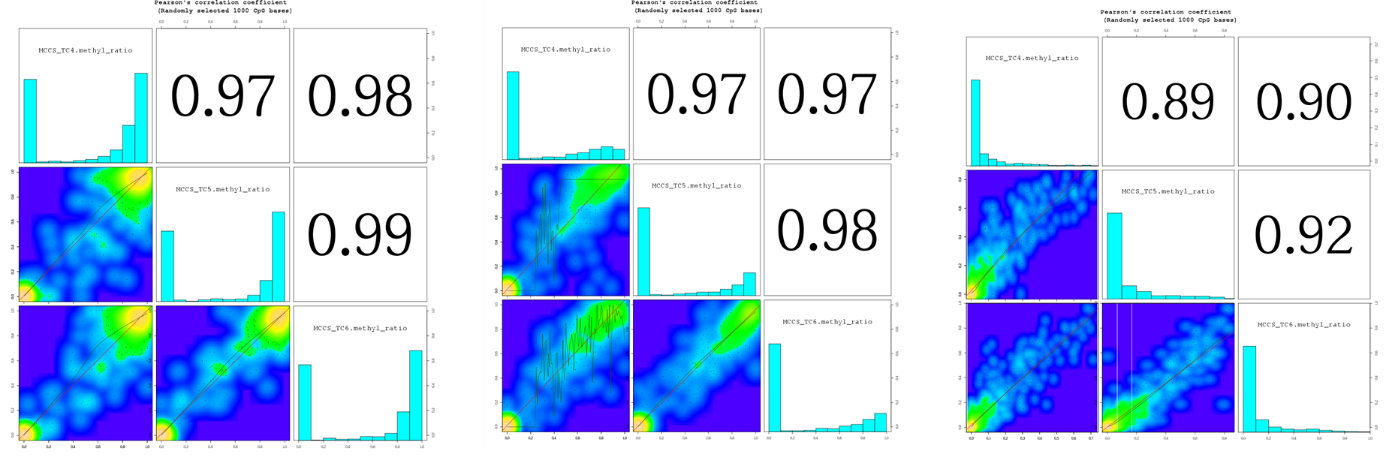


| A | B | C |
| --- | --- | --- |

**Supplementary Figure S5** Plotted the scatter plot & level plot to see the degree of reproducibility between samples using Pearsons’s correlation.  (Range: -1≤ r ≤ 1). CpG context (A). CHG context (B). CHH context (C). ZE (MCCS_TC4), Normal SE (MCCS_TC5) and Abnormal SE (MCCS_TC6).


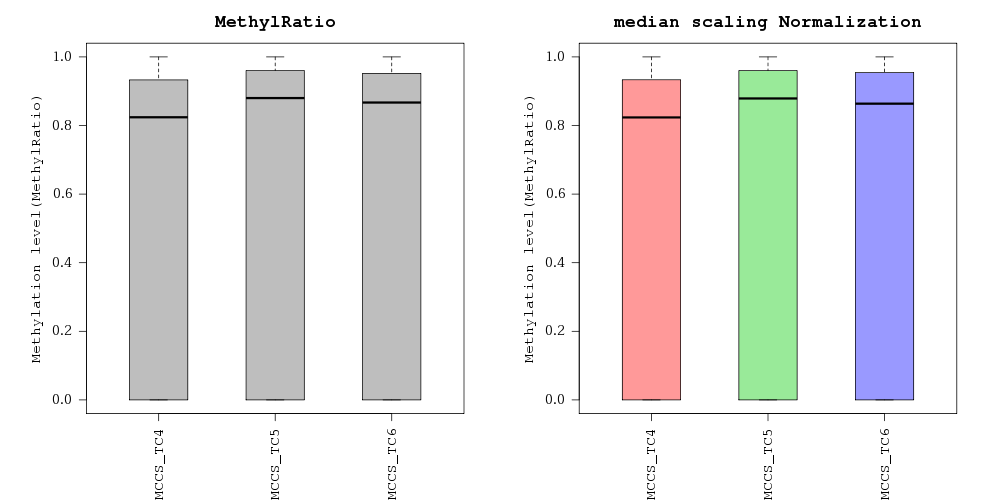


**A**

**
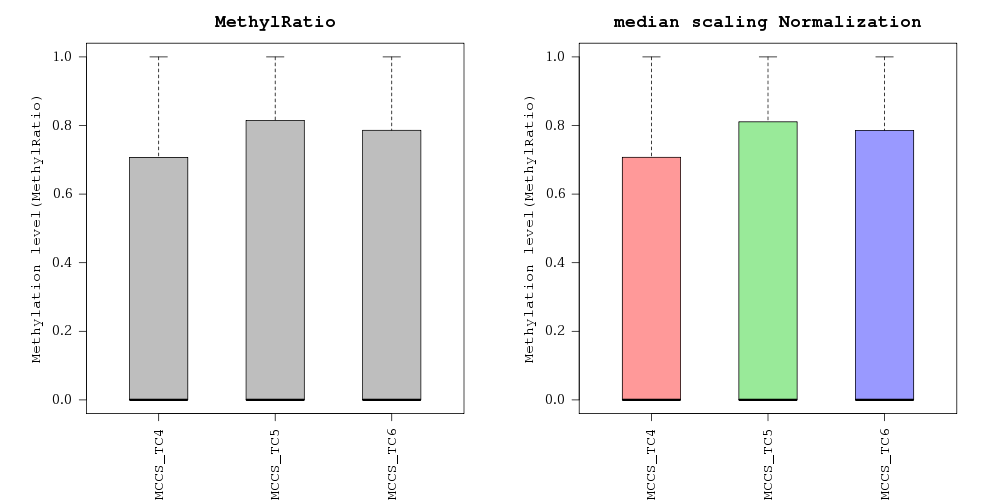
**

B

**
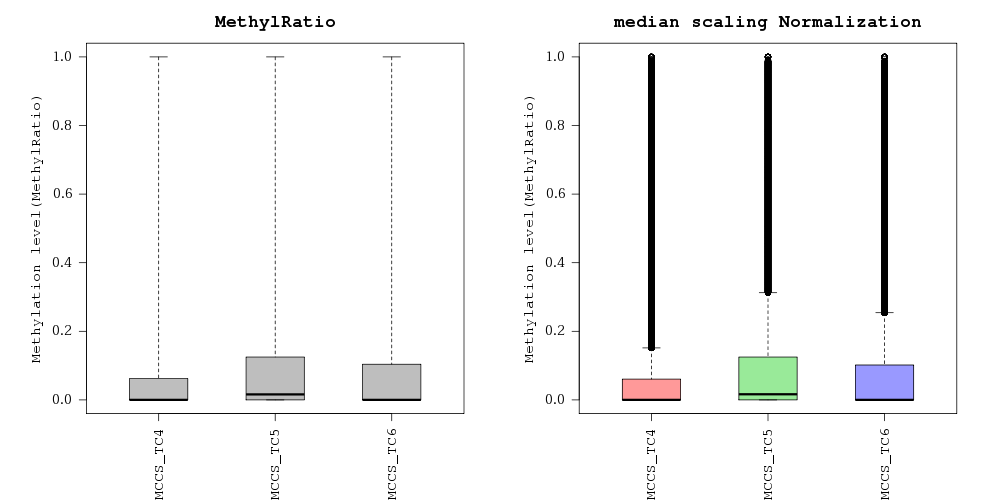
**

**C**

**Supplementary Figure S6** Plotted the box plot to compare distribution of normalized methyl ratio before and after median scaling normalization. The smallest observation (minimum), lower quartile, median, upper quartile, and the largest observation (maximum). CpG context (A). CHG context (B). CHH context (C). ZE (MCCS_TC4), Normal SE (MCCS_TC5) and Abnormal SE (MCCS_TC6).


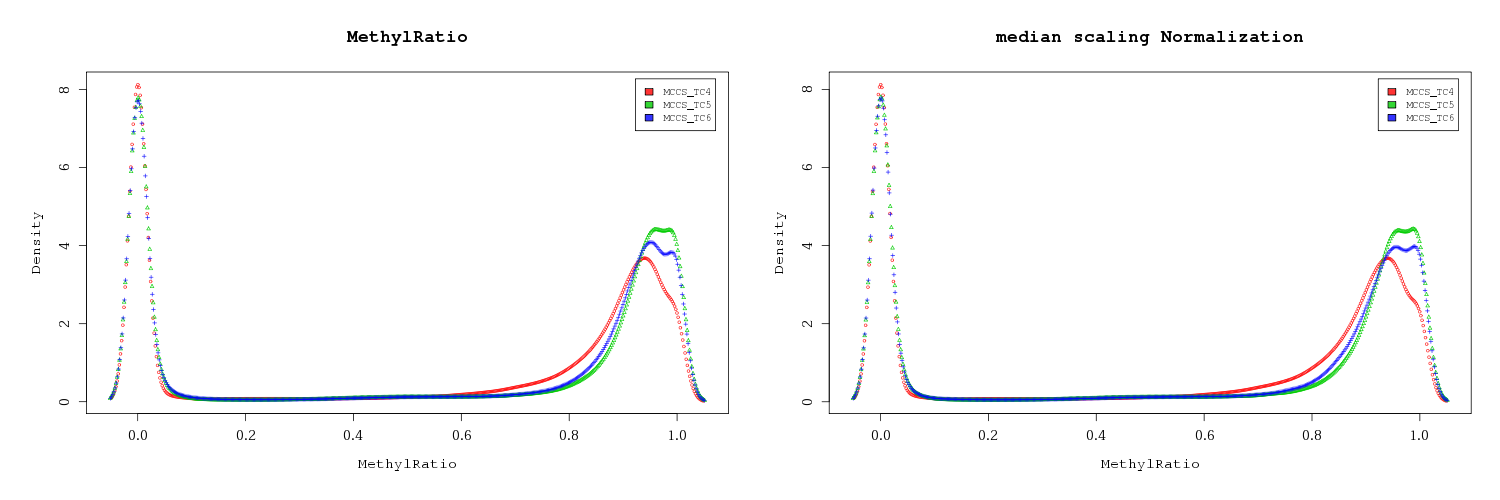


**A**

**
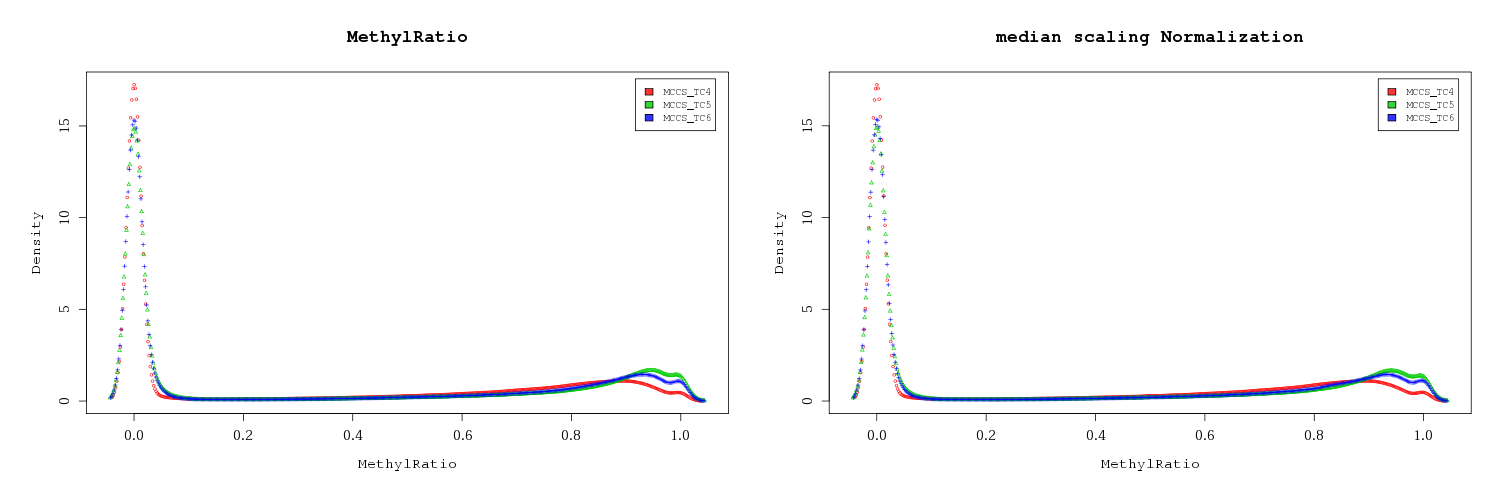

B**

**
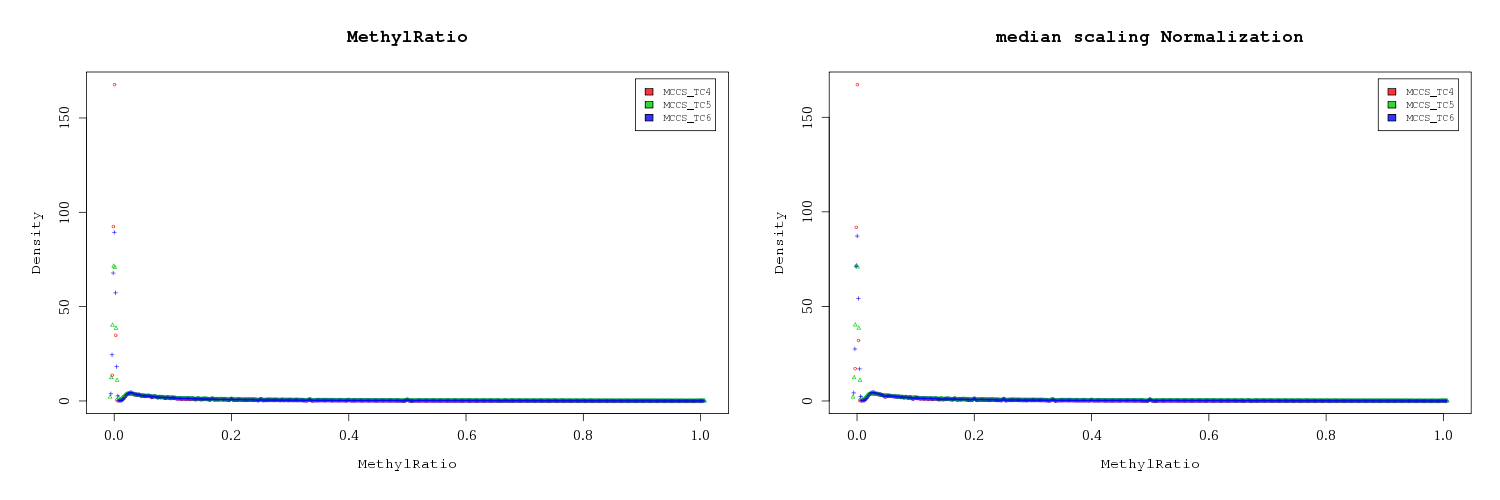
**

**C**

**Supplementary Figure S7** Plotted the density plot to compare distribution of MethylRatio before and after normalization. CpG context (A). CHG context (B). CHH context (C). ZE (MCCS_TC4), Normal SE (MCCS_TC5) and Abnormal SE (MCCS_TC6).

| 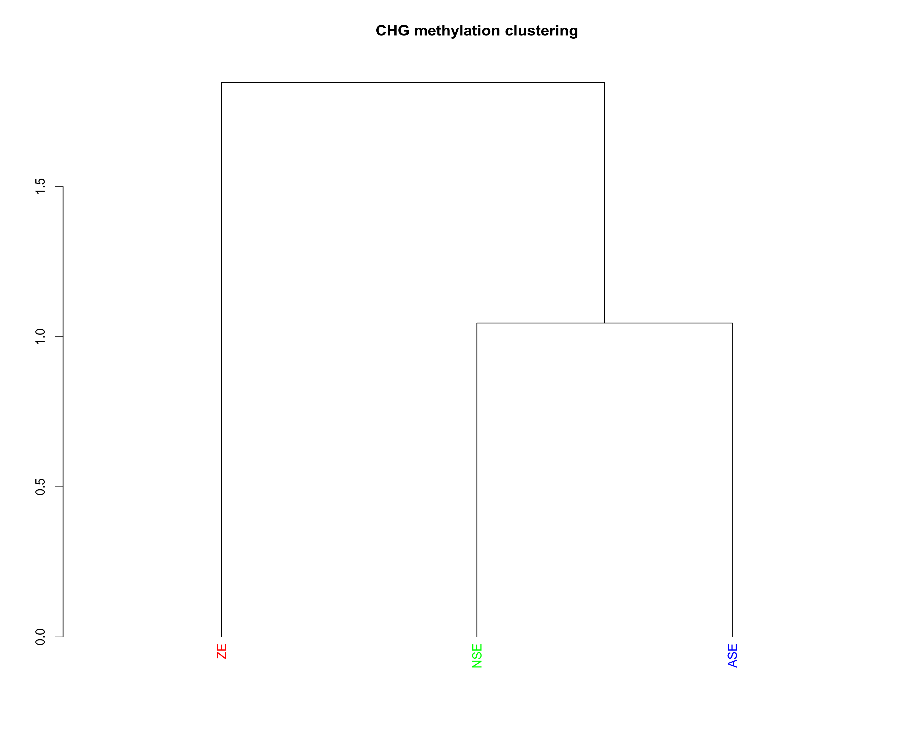  A | 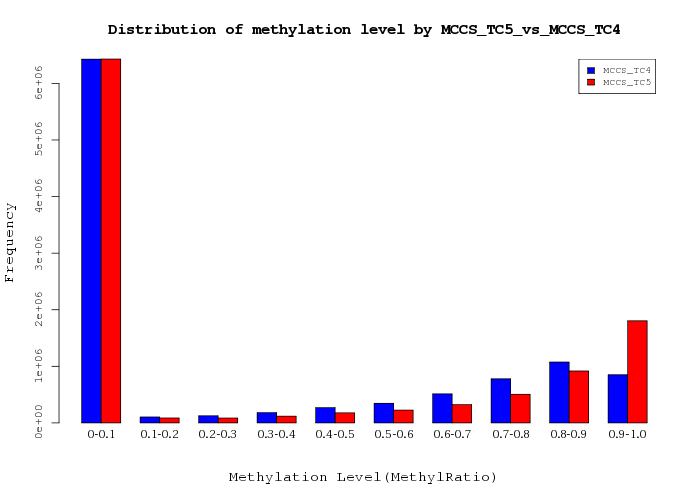  B |
| --- | --- |
| 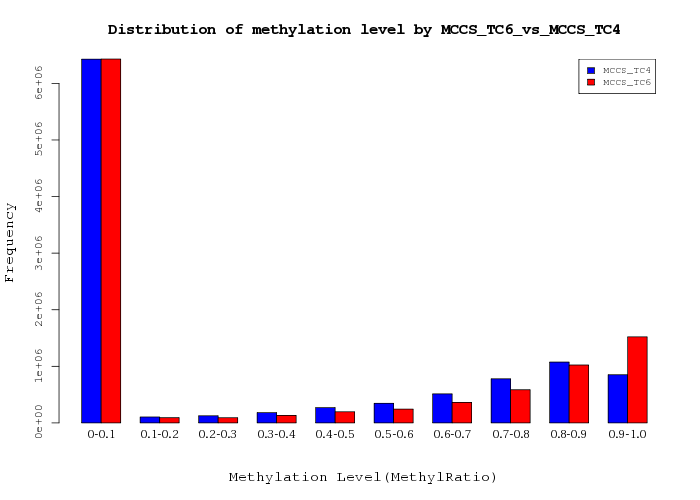  C | 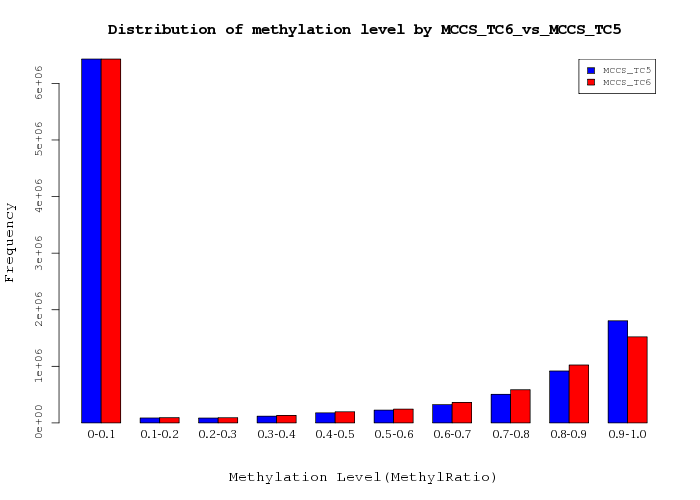  D |

**Supplementary Figure S8.** Methylation distribution analysis in the CHG context of ZE, normal and abnormal SE by WGBS. (A) Hierarchical clustering of the samples by Euclidian distances, where normal and abnormal SE were grouped in the same cluster while ZE was grouped separately showing the similarities between samples in the methylation profile. (B) Frequency bar chart of methylation levels between ZE and normal SE where the highest frequencies were in regions with low levels of methylation for both samples. (C) Same analysis was done for ZE and abnormal SE. (D) Same analysis was done for normal and abnormal SE, showing the same behaviorin; Color code blue (ZE) and red (Normal SE) in B; blue (ZE) and red (abnormal SE) in C; blue (normal SE) and red (abnormal SE) in D. Codes in ZE = (MCCS_TC4), Normal SE = (MCCS_TC5) and Abnormal SE = (MCCS_TC6).

| 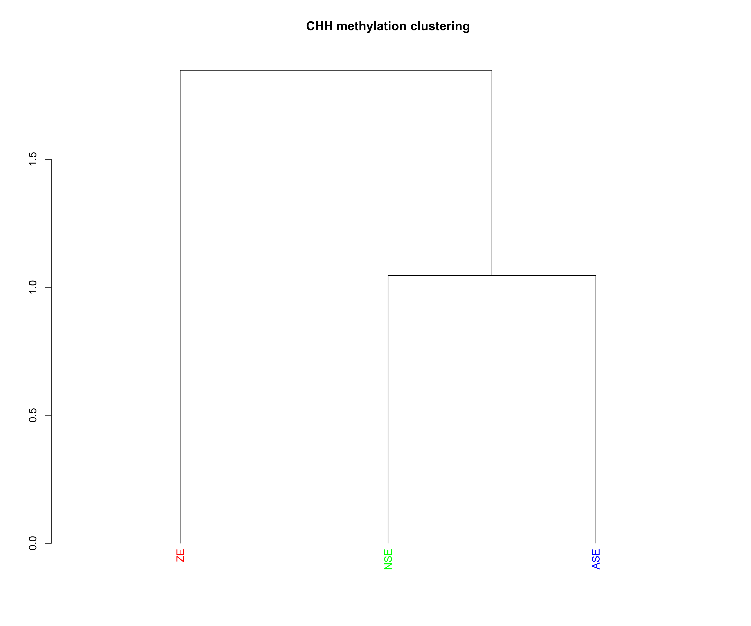  A | 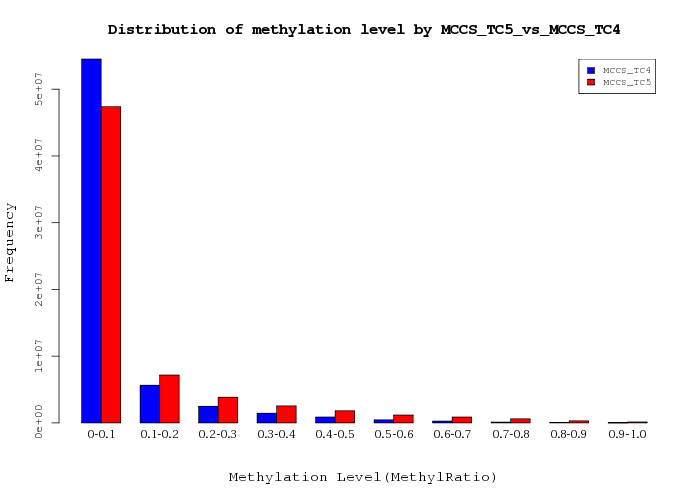  B |
| --- | --- |
| 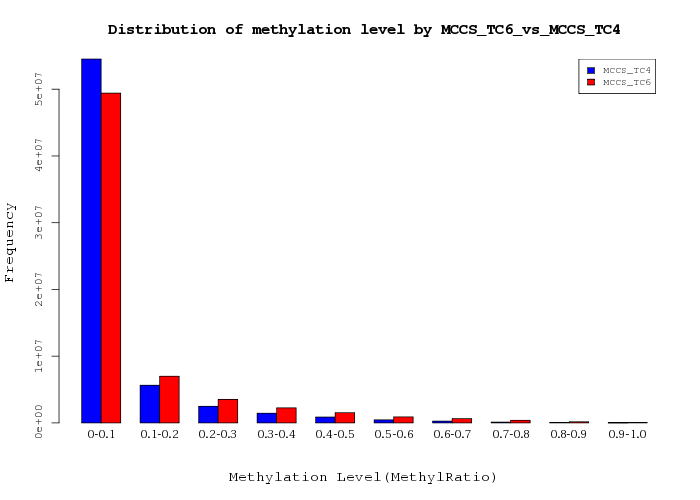  C | 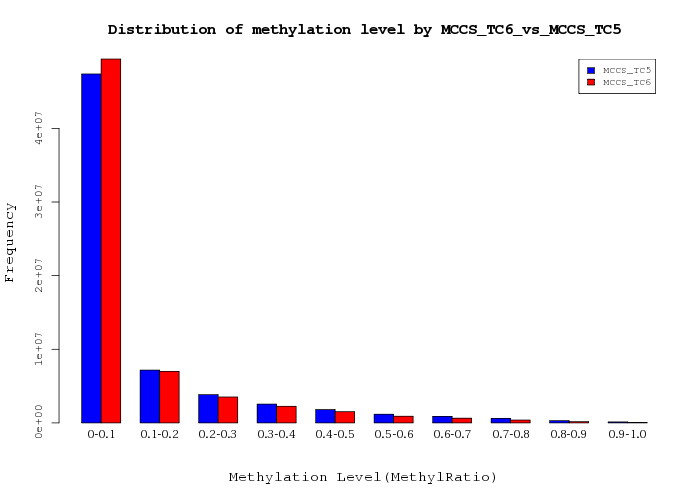  D |
| 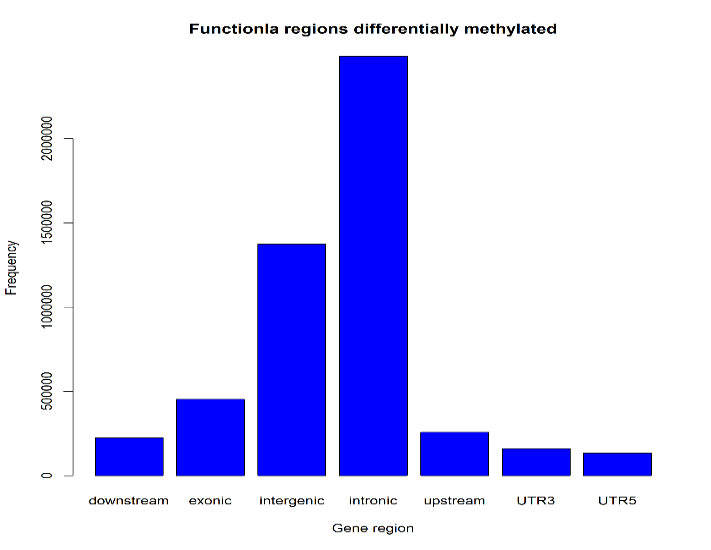  E | 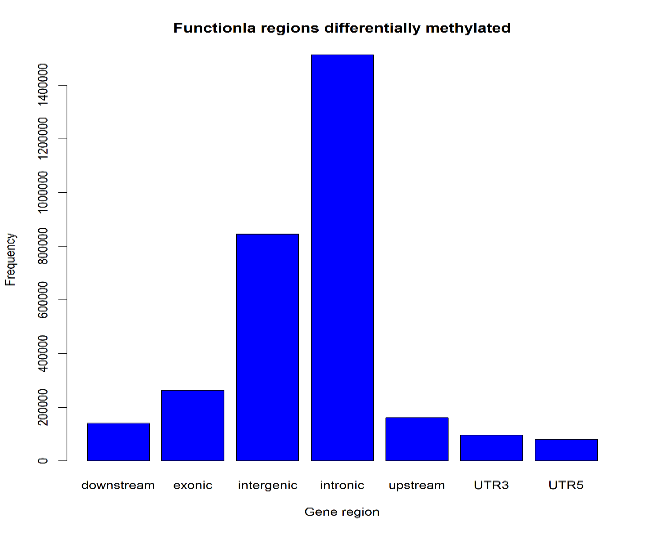  F |
| 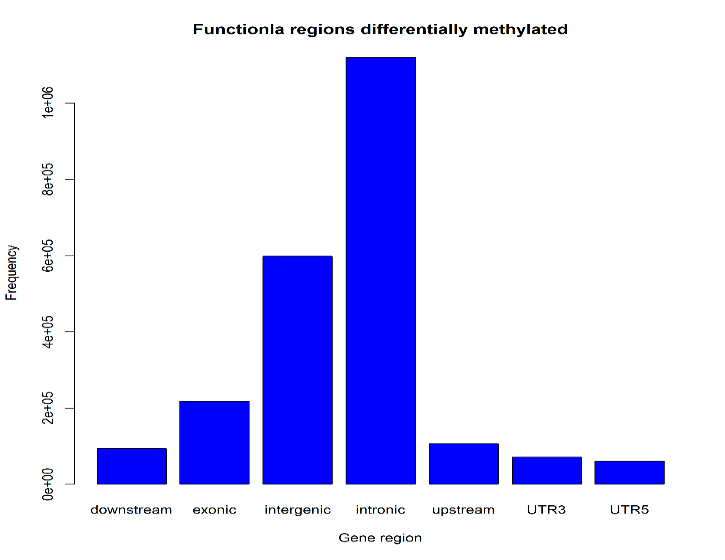  G |  |

**Supplementary Figure S9.** Methylation distribution analysis in the CHH context of the ZE, normal and abnormal SE by WGBS. (A) Hierarchical clustering of the samples by Euclidian distances, where normal and abnormal SE were grouped in the same cluster while ZE were grouped separately showing the similarities between samples in the methylation profile. (B) frequency Histogram of methylation levels between ZE and normal SE where the highest frequencies were in regions hypomethylated for both samples. (C) same analysis was done for ZE and abnormal SE (D) and between normal and abnormal SE showing the same behavior. (E) Frequency bar chart representing the functional regions differentially methylated between ZE and normal SE, (F) ZE and abnormal SE and (G) normal and abnormal SE where intronic regions get the highest frequencies. Color code blue (ZE) and red (Normal SE) in B; blue (ZE) and red (abnormal SE) in C; blue (normal SE) and red (abnormal SE) in D. Codes in ZE = (MCCS_TC4), Normal SE = (MCCS_TC5) and Abnormal SE = (MCCS_TC6).

| 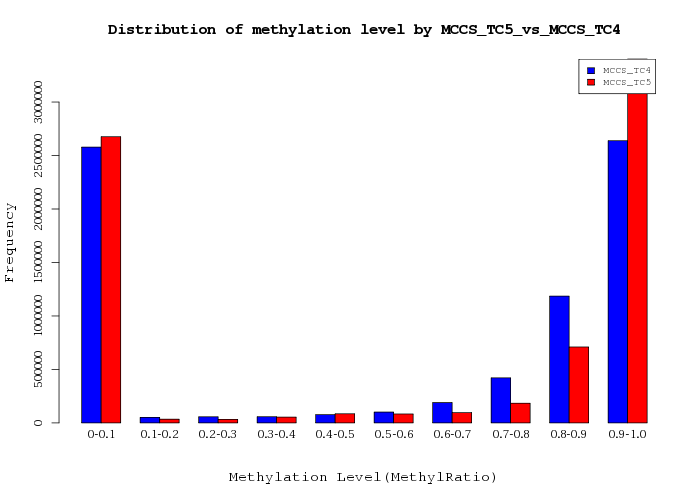  A | 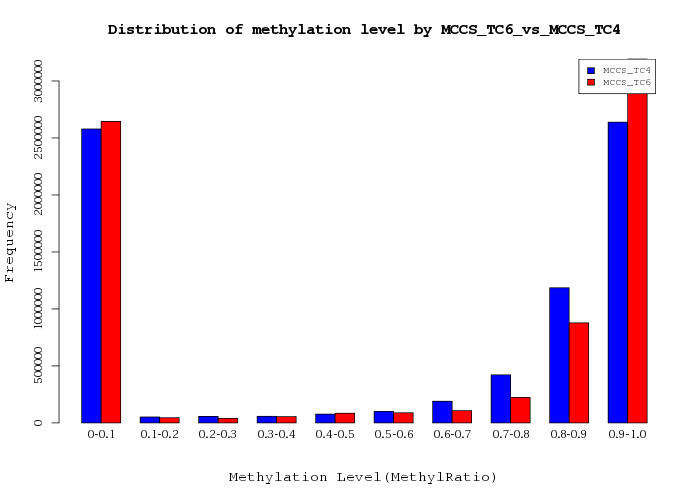  B |
| --- | --- |
| 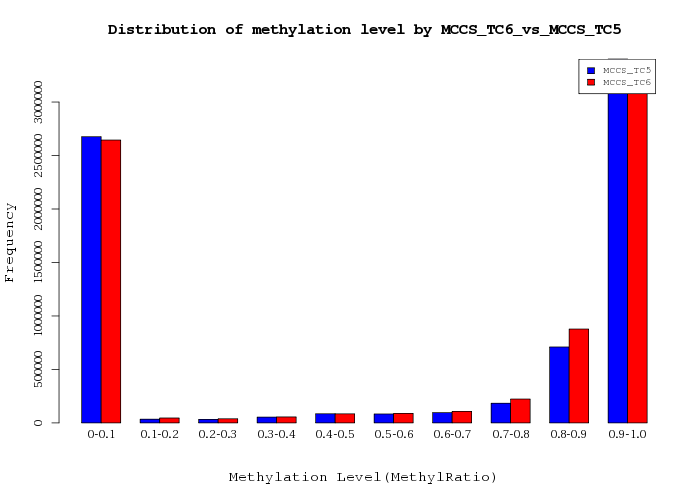  C | **Supplementary Figure S10**. Methylation distribution analysis in the CpG context of ZE, normal and abnormal SEs by WGBS. (A) Frequency bar chart of methylation levels between ZE and normal SE shows bimodal distribution for both samples. (B) Same analysis was done for ZE and abnormal SE (C) and between normal and abnormal SE showing the same bimodal distribution. Color code blue (ZE) and red (Normal SE) in B; blue (ZE) and red (abnormal SE) in C; blue (normal SE) and red (abnormal SE) in D. Codes in ZE = (MCCS_TC4), Normal SE = (MCCS_TC5) and Abnormal SE = (MCCS_TC6). |
|  |  |

**Table 1**. Raw data stats

| Sample ID | Total read bases | Total reads | | GC(%) | | Q20(%) | | Q30(%) |
| --- | --- | --- | --- | --- | --- | --- | --- | --- |
| MCCS_TC4 | 57,203,100,612 | 453,992,862 | | 21.04 | | 91.16 | | 86.14 |
| MCCS_TC5 | 74,059,407,828 | 587,773,078 | | 24.88 | | 76.70 | | 67.08 |
| MCCS_TC6 | 72,546,394,788 | 575,765,038 | | 24.31 | | 76.90 | | 68.30 |
| Total read bases : Total number of bases sequenced | | | | |  | |  |  |
| Total reads : Total number of reads | | |  | |  | |  |  |
| GC(%) : GC content | | |  | |  | |  |  |
| Q20(%) : Ratio of reads that have Phred quality score over 20 | | | | | | |  |  |
| Q30(%) : Ratio of reads that have Phred quality score over 30 | | | | | | |  |  |

**Table 2.** Mean Coverage

| Sample ID | Mean Coverage(X) | Standard Deviation |
| --- | --- | --- |
| MCCS_TC4 | 86.58 | 452.18 |
| MCCS_TC5 | 104.03 | 322.33 |
| MCCS_TC6 | 97.32 | 332.5 |

**Table 3.** Mapping Data Stats

| Sample ID | # of trimmed read bases (bp) | | Average throughput depth of reference genome (X) | | # of uniquely mapped reads (% out of trimmed reads) | # of uniquely mapped reads (% out of trimmed reads) | | Deduplicated reads (deduplicated by Picard tools, % out of mapped reads) | Analyzed reads in BSMAP methylation calling | |  |  |
| --- | --- | --- | --- | --- | --- | --- | --- | --- | --- | --- | --- | --- |
| MCCS_TC4 | 53,854,061,680 | | 162.9 | | 236,539,548 (53.71%) | 45,085,836 (10.24%) | | 203,071,734 (85.85%) | 203,071,419 | |  |  |
| MCCS_TC5 | 57,082,774,038 | | 172.7 | | 290,502,900 (59.96%) | 42,628,118 (8.8%) | | 263,500,348 (90.7%) | 263,500,094 | |  |  |
| MCCS_TC6 | 54,563,049,498 | | 165.0 | | 273,468,040 (59.04%) | 40,003,286 (8.64%) | | 245,099,534 (89.63%) | 245,099,294 | |  |  |
| 1. Sample ID : Sample name. | | | | |  | | |  | | |  | |
| 2. # of trimmed read bases (bp): Total number of bases after trimming. | | | | | | | | | | |  | |
| 3. Average throughput depth of reference genome: Calculated by # of trimmed read bases / | | | | | | | | | | | | |
| reference genome size (ex. human reference size : 3,095,693,983). | | | | | | | | | | |  | |
| 4. # of uniquely mapped reads (% out of trimmed reads): Total uniquely mapped read count, | | | | | | | | | | | | |
| percentage of uniquely mapped reads out of trimmed reads. | | | | | | | | | | |  | |
| 5. # of suppressed non-unique mapped reads: Reads suppressed by multiple mapping , | | | | | | | | | | |  | |
| percentage of non-unique mapped reads out of trimmed reads. | | | | | | | | | | |  | |
| 6. Deduplicated reads (%): Reads after removing PCR duplicates, percentage of deduplicated | | | | | | | | | | | | |
| reads out of uniquely mapped reads. | | | | |  | | |  | | |  | |
| 7. Analyzed reads in BSMAP methylation calling: Used reads that used to extract methylation | | | | | | | | | | | | |
| call for each locus. | | | | |  | | |  | | |  | |
|  | | |  | |  | | |  | | |  | |

**Table 4.** Methylated coverage for each of the three-sequence contexts (CG, CHG, and CHH)

| Methylated coverage in CpG | | | |
| --- | --- | --- | --- |
| Sample ID | Total Coverage in CpG | Methylated coverage in CpG | Methyl % |
| MCCS_TC4 | 360,121,653 | 180,559,166 | 50.14% |
| MCCS_TC5 | 335,893,165 | 181,227,160 | 53.95% |
| MCCS_TC6 | 304,533,349 | 156,477,083 | 51.38% |
| Methylated coverage in CHG | | | |
| Sample ID | Total Coverage in CHG | Methylated coverage in CHG | Methyl % |
| MCCS_TC4 | 448,950,657 | 124,407,306 | 27.71% |
| MCCS_TC5 | 450,008,195 | 142,115,982 | 31.58% |
| MCCS_TC6 | 412,936,021 | 116,566,932 | 28.23% |
| Methylated coverage in CHH | | | |
| Sample ID | Total Coverage in CHH | Methylated coverage in CHH | Methyl % |
| MCCS_TC4 | 2,637,872,887 | 140,961,140 | 5.34% |
| MCCS_TC5 | 2,694,615,611 | 267,030,237 | 9.91% |
| MCCS_TC6 | 2,576,053,329 | 208,904,441 | 8.11% |
